# Predicting β-lactam susceptibility from the genome of *Streptococcus pneumoniae* and other mitis group streptococci

**DOI:** 10.3389/fmicb.2023.1120023

**Published:** 2023-03-02

**Authors:** Helle Brander Eriksen, Kurt Fuursted, Anders Jensen, Christian Salgård Jensen, Xiaohui Nielsen, Jens Jørgen Christensen, Patricia Shewmaker, Ana Rita Rebelo, Frank Møller Aarestrup, Kristian Schønning, Hans-Christian Slotved, the One Day in Denmark (ODiD) Consortium

**Affiliations:** ^1^Department of Clinical Microbiology, Herlev and Gentofte Hospital, Herlev, Denmark; ^2^Department of Bacteria, Parasites, and Fungi, Statens Serum Institut, Copenhagen, Denmark; ^3^Department of Clinical Microbiology, Sygehus Lillebælt, Vejle, Denmark; ^4^Department of Clinical Microbiology, Rigshospitalet, Copenhagen, Denmark; ^5^The Regional Department of Clinical Microbiology, Slagelse, Denmark; ^6^Department of Clinical Medicine, University of Copenhagen, Copenhagen, Denmark; ^7^Centers for Disease Control and Prevention, Atlanta, GA, United States; ^8^Research Group for Genomic Epidemiology, National Food Institute, Technical University of Denmark, Kongens Lyngby, Denmark

**Keywords:** penicillin-binding proteins, penicillin, genotypic susceptibility, pneumococcus, *Streptococcus*

## Abstract

**Introduction:**

For *Streptococcus pneumoniae*, β-lactam susceptibility can be predicted from the amino acid sequence of the penicillin-binding proteins PBP1a, PBP2b, and PBP2x. The combination of PBP-subtypes provides a PBP-profile, which correlates to a phenotypic minimal inhibitory concentration (MIC). The non-*S. pneumoniae* Mitis-group streptococci (MGS) have similar PBPs and exchange *pbp*-alleles with *S. pneumoniae*. We studied whether a simple BLAST analysis could be used to predict phenotypic susceptibility in Danish *S. pneumoniae* isolates and in internationally collected MGS.

**Method:**

Isolates with available WGS and phenotypic susceptibility data were included. For each isolate, the best matching PBP-profile was identified by BLAST analysis. The corresponding MICs for penicillin and ceftriaxone was retrieved. Category agreement (CA), minor-, major-, and very major discrepancy was calculated. Genotypic-phenotypic accuracy was examined with Deming regression.

**Results:**

Among 88 *S. pneumoniae* isolates, 55 isolates had a recognized PBP-profile, and CA was 100% for penicillin and 98.2% for ceftriaxone. In 33 *S. pneumoniae* isolates with a new PBP-profile, CA was 90.9% (penicillin) and 93.8% (ceftriaxone) using the nearest recognized PBP-profile. Applying the *S. pneumoniae* database to non-*S. pneumoniae* MGS revealed that none had a recognized PBP-profile. For *Streptococcus pseudopneumoniae*, CA was 100% for penicillin and ceftriaxone in 19 susceptible isolates. In 33 *Streptococcus mitis* isolates, CA was 75.8% (penicillin) and 86.2% (ceftriaxone) and in 25 *Streptococcus oralis* isolates CA was 8% (penicillin) and 100% (ceftriaxone).

**Conclusion:**

Using a simple BLAST analysis, genotypic susceptibility prediction was accurate in Danish *S. pneumoniae* isolates, particularly in isolates with recognized PBP-profiles. Susceptibility was poorly predicted in other MGS using the current database.

## 1. Introduction

With increased use of whole genome sequencing (WGS) in clinical microbiological laboratories comes the need for developing robust methods for the prediction of antimicrobial susceptibility from WGS data and for generating data for phenotypic-genotypic correlation of susceptibility (Ellington et al., 2017). Genotypic susceptibility prediction from WGS data is of value in culture-negative specimens, e.g., due to prior antibiotic treatment. *Streptococcus pneumoniae* is a major pathogen which in 2019 caused 639 cases of invasive infections in Denmark, primarily bloodstream infections (BSI) and meningitis (DANMAP, 2019). The 30-day mortality for *S. pneumoniae* BSIs has been estimated to 16% (Christensen et al., 2012). Of other bacterial species among the Mitis group streptococci (MGS), *Streptococcus mitis* and *Streptococcus oralis* are mainly commensals, but have significant clinical importance, e.g., in infective endocarditis (Rasmussen et al., 2016). *Streptococcus pseudopneumoniae* is mainly associated with lower respiratory tract infections (Arbique et al., 2004) and hepatic/bile-duct infections (Fuursted et al., 2016).

Most invasive Danish *S. pneumoniae* are susceptible toward benzylpenicillin, 95.1% in 2019 (DANMAP, 2019), and this is the recommended treatment for susceptible strains. Benzylpenicillin is also the recommended treatment for MGS endocarditis (Habib et al., 2009). Penicillin binds to the transpeptidase domain (TPD) of the penicillin-binding-protein (PBP) and inhibits cell-wall synthesis (Hakenbeck et al., 2012). Penicillin non-susceptible strains have mutations in the *pbp2x* and *pbp2b* alleles, that are associated with low-level resistance, and alterations in the *pbp1a* allele is associated with high-level resistance (Smith and Klugman, 1998). While susceptible *S. pneumoniae* have conserved *pbp*-alleles, alleles of non-susceptible strains have a mosaic structure due to horizontal gene transfer by homologous recombination with alleles from non-susceptible MGS. Both susceptible and non-susceptible isolates of *S. mitis, S. oralis* and *S. infantis* have a considerable number of polymorphic sites in all three *pbp-genes*, which is an important reservoir for pneumococcal resistance genes (Jensen et al., 2015). In *S. pseudopneumoniae*, penicillin-susceptible isolates contain pbp2x alleles distinct from *S. pneumoniae* and *S. mitis*, while penicillin-resistant isolates display similar mosaic structures (Van Der Linden et al., 2017).

A method for genotypic prediction of β-lactam susceptibility in *S. pneumoniae* was first developed by Metcalf et al. (2016b). From a collection of invasive *S. pneumoniae* isolates, the amino acid sequences of the TPD of the three PBPs, PBP1a, PBP2b and PBP2x, were characterized, and subsequently 69, 77 and 127 unique initial subtypes were identified. A “PBP-profile” could be assigned as a combination of the three PBP-TPD-subtypes, and each PBP-profile correlated to phenotypic MIC values for penicillin and other β-lactam antibiotics. The method was further validated by Metcalf et al. (2016a) and refined by Li et al. (2016) to include newly encountered PBP profiles. Using statistical predictive models, susceptibility could accurately be predicted in 94–99% of cases (Li et al., 2016). This method was also used in isolates with previously uncharacterized PBP-profiles, and showed an overall essential agreement of >97% and a category agreement >93% (Li et al., 2017).

In the present study, we examined an alternative method for genomic susceptibility prediction, which require only basic bioinformatic skills. We used a simple BLAST analysis to identify the nearest PBP-profile of an isolate and used the correlated phenotypic MIC for susceptibility prediction.

We examined the performance of this method in a collection of Danish *S. pneumoniae* isolates, both with recognized or new PBP-profiles. Since non-*S. pneumoniae* MGS have similar PBPs and exchange alleles with *S. pneumoniae*, we were curious whether the same method could be applied to these species and how well susceptibility could be predicted.

## 2. Materials and methods

### 2.1. Strains

We included 88 clinical *S. pneumoniae* isolates, 19 *S. pseudopneumoniae* isolates, 33 *S. mitis* isolates, 25 *S. oralis* isolates and 1 *S. infantis* isolate based on available WGS data and phenotypic susceptibility (MIC) for penicillin and for most isolates ceftriaxone (CFT). Isolates were retrieved from Danish and international collections of MGS (Supplementary Table S1): (1) Danish laboratory surveillance system at the Danish national *Neisseria* and *Streptococcus* Reference Laboratory (NSR), Statens Serum Institut, Denmark (SSI) (Kavalari et al., 2019), (2) Department of Biomedicine, Faculty of Health, Aarhus University, Denmark (Aarhus) (Jensen et al., 2015, 2016), (3) The Regional Department of Clinical Microbiology, Region Zealand, Slagelse Hospital, Denmark (Slagelse) (Rasmussen et al., 2016), (4) Queen Elizabeth II Health Sciences Center, Halifax, Canada *via* the Centers for Disease Control and Prevention, Atlanta, GA, United States (CDC) (Arbique et al., 2004), and (5) the One Day in Denmark (ODiD) project, National Food Institute, Technical University of Denmark, Denmark (Rebelo et al., 2022).

### 2.2. WGS, species identification, multi locus sequence typing (MLST) and molecular serotyping

Isolates from SSI and the 10 *S. pseudopneumoniae* isolates from CDC were sequenced by paired-end Illumina sequencing (Illumina MiSeq) as previously described (Kavalari et al., 2019). For some isolates from Slagelse and all isolates from Aarhus, WGS data were retrieved from GenBank.^1^ The remaining isolates from Slagelse were sequenced using Illumina HiSeq 2000 as previously described (4). Isolates from the ODiD-project were sequenced using the Illumina NextSeq 500 platform and pair-end sequencing and The Center for Genomic Epidemiology pipeline (Rebelo et al., 2022). Species identification was confirmed by cgMLSA from WGS data (Jensen et al., 2021). Software Pathogenwatch (Wellcome Sanger Institute)^2^ was used for MLST and molecular serotyping of *S. pneumoniae* isolates. For MLST, the seven housekeeping genes *aroE, gdh, gki, recP, spi*, *xpt and ddl* were used, retrieved from the PubMLST website https://pubmlst.org/spneumoniae/ (Jolley et al., 2018). The method used for serotyping is based on SeroBA (Epping et al., 2018).

### 2.3. Phenotypic susceptibility testing

For the isolates from Slagelse/CDC, the ODiD-project and from SSI from year 2010, the MIC for penicillin and ceftriaxone was determined using Sensititre broth microdilution method (*Streptococcus* species MIC Plate, STP6F, Trek Diagnostic System, United Kingdom) according to manufacturer’s instructions. Before year 2010, the isolates from SSI was tested with Etest (AB Biodisk, Solna, Sweden) on Danish Blood Agar (Resistance plates, SSI Diagnostica) incubated at 36° C, 5% CO_2_ (DANMAP, 2009). Isolates from Aarhus, were tested with agar dilution method (Jensen et al., 2015). EUCAST breakpoints table version 10^3^ was used for interpretation of SIR susceptibility.

### 2.4. Prediction of genotypic β-lactam susceptibility

Genotypic prediction of β-lactam susceptibility was performed using the classification system described by Li et al. (2016). From the CDC *Streptococcus* laboratory website,^4^ we obtained the amino acid sequences for the TPD subtypes of PBP1a, PBP2b and PBP2x in *S. pneumoniae* which at the time of data analysis was 101, 121, and 203 subtypes of PBP1a, PBP2b and PBP2x (June 2017). Using WGS data from our isolates, a nucleotide-protein BLAST analysis was performed using the NCBI Genome Workbench, version 3.0.0.^5^ to identify the nearest PBP1a, PBP2b and PBP2x subtypes. In the case of 100% identity for all three PBPs, a “PBP-profile” (PBP-type) was assigned, and the corresponding phenotypic MIC values for penicillin and ceftriaxone was retrieved from the available “PBP-type-To-MIC table,” accessed in June 2017. This contains 422 PBP-profiles for penicillin and 317 PBP-profiles for ceftriaxone. Although the number of PBP subtypes has increased significantly since (477 subtypes of PBP1a, 658 subtypes of PBP2b and 1050 subtypes of PBP2x by September 2022), the correlation of a whole PBP-type to a phenotypic susceptibility has not been updated since.

Isolates without a recognized PBP-profile included isolates where the exact combination of the three PBP-TPBs was not in the PBP-to-MIC table. Other isolates, particularly non-*S. pneumoniae* MGS, had substitutions in one or more PBP-TPDs. For MIC-prediction in these isolates, we created a database containing the concatenated PBP-TPD sequence for all published PBP-profiles with a correlating MIC. This database was used for a BLAST analysis with the concatenated PBP-TPD sequence for a given isolate. The best matching PBP-profile was identified as the result with the highest percent identity together with the highest total BLAST score.

Using this method, isolates could have a PBP-profile as best match with more substitutions than the sum of substitutions in each PBP separately. The applied method was chosen, because it requires only basic bioinformatic skills, a simple database containing the PBP-TPD amino acid sequences and the correlating phenotypic MIC and is fast to run.

### 2.5. Phylogenetic analysis of the concatenated PBP TPD sequence

A phylogenetic analysis of the concatenated PBP amino acid sequence from each isolate was performed using IQ-TREE (Nguyen et al., 2015) with a Blosum62 scoring matrix for amino-acid substitutions. The model only allowed amino acid sequences of equal length. All, except three *S. mitis* isolates, had a concatenated sequence of 914 amino acids. Isolates *Sm7* and *Sm19* had one insertion (position 326) and *Sm32* three insertions (position 326–328), which were omitted for the phylogenetic analyses after ensuring that the PBP-profile was the same before and after the modification. We included the concatenated PBP amino acid sequence of the species type strains: *S. pneumoniae* NCTC 7465^T^ (NCBI reference NZ_LN831051.1), *S. pseudopneumoniae* ATCC-BAA-960^T^ (NZ_AICS00000000.1), *S. mitis* NCTC 12261^T^ (NZ_CP028414.1), *S. oralis* ATCC 35037^T^ (NZ_LR134336.1) and *S. infantis* ATCC 700779^T^ (NZ_GL732439.1). An outlier reference sequence was included: the concatenated PBP-sequence of *Streptococcus dysgalactiae subspecies equisimilis* AC2713 (NC_019042.1) was modified to a length of 914 amino acids by using an alignment with *S. pneumoniae* R6 to omit insertions at positions 525, 526, 532, 533, 650, 692, 897, and deletions at positions 327, 328, 867 were replaced by the corresponding amino acid in R6. The software iTOL version 5.6.2^6^ was used for visualization of trees.

### 2.6. Statistical analyses

We reported the number of isolates phenotypic and genotypic susceptible (S), susceptible-increased-exposure (I) and resistant (R) toward penicillin and ceftriaxone. Category agreement (CA) was defined as correctly predicted S-I-R, minor discrepancy (MiD) was susceptible isolates predicted to be susceptible-increased-exposure, susceptible-increased-exposure isolates predicted to be resistant and opposite. Major discrepancy (MaD) was isolates being resistant by genotype but susceptible by phenotype, while very major discrepancy (VMaD) was isolates being susceptible by genotype but resistant by phenotype. Essential agreement (EA) was an equal genotypic/ phenotypic MIC +/− one two-fold dilution. For isolates with an MIC ≤0.03, the value 0.03 was used.

For *S. pneumoniae* positive and negative predictive values (PPV, NPV) for non-susceptibility were calculated. Deming regression models were used to visualize log2 transformed phenotypic-genotypic MIC, using statistical software RStudio version 1.1.453. Isolates with an MIC ≤0.03, was given the value 0.03 and MIC >2 the value 4.

### 2.7. Ethical considerations

The data used did not include any personalized data.

## 3. Results

We included 88 isolates of *S. pneumoniae*, of which 62 were from BSI or cerebrospinal fluid (CSF), and the remaining isolates were mainly from respiratory specimens. *S. pneumoniae* isolates were collected in Denmark between 1999 and 2018 except one historic isolate (1943), and included 23 different serotypes and 34 different MLSTs. The 19 *S. pseudopneumoniae* isolates included 13 respiratory isolates, of which most originated from Canada (5). Among the 33 *S. mitis* isolates, 14 were from BSI and the remaining were mainly respiratory isolates and among the 25 *S. oralis* isolates, 21 were BSI, mainly endocarditis isolates. The *S. infantis* isolate was isolated from a urogenital infection (Supplementary Table S1). All species were identified using cgMLSA (Supplementary Figure S1).

### 3.1. PBP-types in Danish *Streptococcus pneumoniae* and MGS

In 55 of 88 *S. pneumoniae* isolates (62.5%), PBP1a, PBP2b and PBP2x all exactly matched a subtype in the database, and a recognized PBP-profile and a corresponding MIC could be predicted. Among isolates from the ODiD-project, 11 of 16 isolates (68.8%) had a recognized PBP-profile.

In the remaining 33 *S. pneumoniae* isolates, we found a new PBP-profile, since that exact combination of PBP-subtypes was not in the database, or there were new substitutions in the PBPs. There was between 1 and 43 substitutions in the concatenated PBP-sequence, when compared to the best matching recognized PBP-profile. (Table 1). Among the 88 *S. pneumoniae* isolates, there were 37 unique PBP-profiles, of which 17 were a recognized PBP-profile.

**Table 1:** Unique PBP-profiles and PBP1a-, PBP2b- and PBP2x-subtypes in *Streptococcus pneumoniae* and MLST and genomic serotypes.

PBP-profile/nearest PPB-profile	Number of isolates	PBP-profile identity %	Substi-tutions	PBP1a		PBP2b		PBP2x			
				Nearest subtype	Substi-tutions	Nearest subtype	Substi-tutions	Nearest subtype	Substi-tuitions	MLST (n)	Serotypes (n)
Type strain NTCT 7465 PT_0–4-0	1	100	0	1a0	0	2b4	0	2×0	0	ST615	1
PT_0–0-0	5	100	0	1a0	0	2b0	0	2×0	0	11,131, 452, 2,964, NA (2)	12F, 24, 35D (3)
PT_0–0-0	1	99.89	1	1a0	0	2b0	1	2×0	0	7,179	24
PT_0–0-0	2	99.23	7	1a86	0	2b82	0	2×162	0	448,1,229	NT
PT_0–0-2	1	100	0	1a0	0	2b0	0	2×2	0	4,753	10A
PT_0–0-28	10	99.12	8	1a2	0	2b0	0	2×28	7	162 (9), NA (1)	24
PT_0–0-3	1	100	0	1a0	0	2b0	0	2×3	0	NA	7C
PT_0–1-1	1	97.48	23	1a0	4	2b1	0	2×99	7	13,224	7C
PT_0–1-2	4	99.34	6	1a2	0	2b103	0	2×0	0	11,100	24
PT_1–0-0	2	100	0	1a1	0	2b0	0	2×0	0	3,811	15A
PT_12–0-0	1	100	0	1a12	0	2b0	0	2×0	0	1,766	31
PT_13–11-33	1	99.78	2	1a13	1	2b11	1	2×33	1	271	19F
PT_13–14-26	1	100	0	1a13	0	2b14	0	2×26	0	320	19A
PT_15–12-18	1	100	0	1a15	0	2b14	0	2×18	0	81	6A
PT-15-14-96	1	97.81	20	1a15	0	2b38	0	2×43	5	NA	6A
PT_15–16-8	1	99.78	2	1a15	0	2b12	0	2×8	0	156	11A
PT_15–7-8	1	98.91	10	1a15	0	2b49	0	2×36	0	166	11A
PT_15–7-8	1	98.36	15	1a15	0	2b76	0	2×36	4	838	9 V
PT_17–15-22	2	100	0	1a17	0	2b15	0	2×22	0	230, NA	24
PT_17–15-22	1	98.03	18	1a17	0	2b12	0	2×22	0	156	9 V
PT_17–15-22	2	97.16	26	1a17	0	2b15	0	2×171	3	4,253	24
PT_17–16-47	1	99.56	4	1a17	0	2b39	0	2×18	0	276	19A
PT_18–7-8	1	97.15	26	1a10	0	2b53	0	2×20	0	NA	21
PT_2–0-0	25	100	0	1a2	0	2b0	0	2×0	0	72 (23), 2,567 (2)	24 (23), 29 (2)
PT_20–18-25	1	98.58	13	1a8	4	2b18	0	2×25	0	275	15B
PT_2–0-2	1	100	0	1a2	0	2b0	0	2×2	0	1,262	15C
PT_2–6-2 / PT_1–6-0	1	99.78	2	1a1	0	2b82	0	2×2	0	2,369	20
PT_27–11-8	1	97.81	20	1a47	1	2b11	0	2×8	2	NA	NT
PT_34–32-43	1	100	0	1a34	0	2b32	0	2×43	0	179	19F
PT_34–89-147	1	99.67	3	1a25	0	2b7	0	2×56	0	8,991	NT
PT_3–6-5	7	100	0	1a3	0	2b6	0	2×5	0	53	8
PT_4–7-7	1	100	0	1a4	0	2b7	0	2×7	0	558	35B
PT_54–61-92	1	95.3	43	1a25	15	2b15	2	2×91	4	NA	NT
PT_62–0-2	3	100	0	1a62	0	2b0	0	2×2	0	NA	3
PT_7–1-1	3	100	0	1a7	0	2b1	0	2×1	0	1,349, 13,087	1, 23B
PT_7–1-30	1	98.91	10	1a7	0	2b1	0	2×123	3	3,369	35D
PT_78–0-0	7	100	0	1a78	0	2b0	0	2×0	0	162	24
PT_8–67–103	1	100	0	1a8	0	2b67	0	2×103	0	135	6B

None of the non-*S. pneumoniae* MGS had a recognized PBP-profile. In *S. pseudopneumoniae*, 5 of 19 isolates had a PBP2b sequence also found in *S. pneumoniae*, (PBP2b0). Nearest PBP-profiles had 11–19 substitutions in the concatenated PBP-sequence (Supplementary Table S2). The 33 *S. mitis* isolates were more diverse in their PBP profiles with no isolate having the exact same amino acid sequence as another isolate. Five isolates had a PBP2b and/or PBP2x-subtype that were recognized from *S. pneumoniae* (Supplementary Table S4). The *S. oralis* isolates generally had more substitutions (40–91) than *S. mitis*. In 22 of the 25 isolates, the nearest PBP-profile was PT_17–1-22, but mostly with different substitutions (Supplementary Table S6). The one *S. infantis* isolate had 94 substitutions (Supplementary Table S8).

### 3.2. Correlation of PBP-profile with MLST and genomic serotype in *Streptococcus pneumoniae*

For six unique PBP-profiles, isolates had several MLSTs, while for six other unique PBP-profiles all isolates with the same profile had the same MLST. Isolates with the same MLST profile could have different PBP-profiles, e.g., ST-162 isolates had both PT_0–0-28 (8 substitutions, 9 isolates) and PT_78–0-0- (7 substitutions, 7 isolates) as the nearest PBP-profile. Most isolates belonged to serogroup 24 but represented eight different PBP-profiles. Likewise, PT_0–0-0 and PT_2–0-0- were seen in more than one serotype (Table 1).

### 3.3. Penicillin: Correlation between genotypic MIC prediction and phenotypic susceptibility

**Figure 1:**
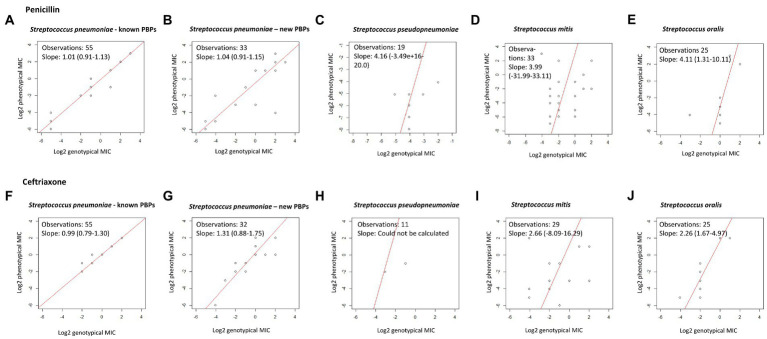
Deming regression of genotypic and phenotypic MIC for penicillin and ceftriaxone. Using log2-transformed genotypic and phenotypic MIC, statistical software R. A slope of 1 indicates exact MIC agreement. **(A–E)** Show results for penicillin. **(F–J)** Show results for ceftriaxone.

Among the 55 *S. pneumoniae* isolates with a recognized PBP-profile, 46 were phenotypic and genotypic susceptible to penicillin, seven were phenotypic and genotypic susceptible-increased-exposure, and two were phenotypic and genotypic resistant, and this resulted in 100% CA (Table 2). EA was 98.2%; one isolate with a genotypic MIC of 2 μg/mL had a phenotypic MIC of 0.5 μg/mL (Table 3). Among the 33 isolates with a new PBP-profile not included in the database, more isolates had reduced susceptibility. CA was 90.9% with a MiD in two isolates and a MaD in one isolate (Table 2), and these three isolates contained many substitutions (23, 26, and 43). EA was 93.9% (Table 4). PPV and NPV for non-susceptibility were 93 and 94%. Genotypic-phenotypic accuracy using Deming regression showed a slope closer to unity for isolates with a recognized PBP-profile [1.01 (0.91–1.13)], compared to isolates with a new PBP-profile [1.04 (0.91–1.15)] (Figures 1A,B).

**Table 2:** Phenotypic-genotypic correlation for penicillin and ceftriaxone susceptibility in *S. pneumoniae* and non-*S. pneumoniae* mitis group streptococci.

	*S. pneumoniae*	*S. pneumoniae*	*S. pseudopneumoniae*	*S. mitis*	*S. oralis*
	Recognized PBP-profiles	New PBP-profiles			
Number of isolates	55	33	19	33	25
PBP-substitutions	0	1–43	11–24	9–66	40–91
**Penicillin susceptibility**					
Genotypic S/I/R ^A^	46/7/2	18/8/7	19/0/0	25/6/2	1/23/1
Phenotypic S/I/R ^A^	46/7/2	18/10/5	19/0/0	27/4/2	23/0/2
Category agreement ^B^	100% (55/55)	90.9% (30/33)	100% (19/19)	75.8% (25/33)	8% (2/25)
Minor discrepancy ^C^	0% (0/63)	5.9% (2/33)	0% (0/19)	18.2% (6/33)	92% (23/25)
Major discrepancy ^D^	0% (0/63)	2.9% (1/33)	0% (0/19)	3.0% (1/33)	0% (0/25)
Very major discrepancy ^E^	0% (0/63)	0% (0/33)	0% (0/19)	3.0% (1/33)	0% (0/25)
**Ceftriaxone susceptibility**					
Genotypic S/I/R/NA ^A^	52/2/1	23/7/2/1	12/0/0	24/0/5/4	23/0/2
Phenotypical S/I/R ^A^	51/3/1	24/7/2	18/0/0	30/0/3	23/0/2
Category agreement ^B^	98.2% (54/55)	93.8% (30/32)	100% (11/11)	86.2% (25/29)	100% (25/25)
Minor discrepancy ^C^	1.8% (1/55)	6.3% (2/32)	0% (0/12)	0% (0/29)	0% (0/25)
Major discrepancy ^D^	0% (0/55)	0% (0/32)	0% (0/12)	10.3% (3/29)	0% (0/25)
Very major discrepancy ^E^	0% (0/55)	0% (0/32)	0% (0/12)	3.4% (1/29)	0% (0/25)

^A^ S/I/R: Susceptible standard dosing regimen/Susceptible increased exposure/Resistant according to EUCAST breakpoints v. 10.0. For *S. pneumoniae*: S: ≤0.06 mg/L (PEN), S ≤ 0.5 mg/L (CFT); I: 0.06–2 mg/L (PEN), 1–2 (CFT); R > 2 (PEN/CFT). For mitis group streptococci (*S. viridans*): S ≤ 0.25 (PEN), S ≤ 0.5 (CFT); I: 0.5–2 (PEN), R > 2 (PEN), R > 0.5 (CFT). ^B^ Category agreement: Same S / I / R category. ^C^ Minor discrepancy: Genotype S and phenotype I or genotype I and phenotype R or opposite. ^D^ Major discrepancy: Genotype R and phenotype S. ^E^ Very major discrepancy: Genotype S and phenotype R.

**Table 3:** Genotypic and phenotypic susceptibility in *Streptococcus pneumoniae* isolates with a recognized PBP-profile.

Isolate	ID	Year	Source of infection	PBP-profile	Substi-tutions	Oxacillin	Penicillin				Ceftriaxone			
						Pheno-typic mm	Geno-typic MIC	Geno-typic S-I-R	Pheno-typic MIC	Pheno-typic S-I-R	Geno-typic MIC	Geno-typic S-I-R	Pheno-typic MIC	Pheno-typic S-I-R
2011–1853	Pn1	2011	Invasive	PT_0–0-0	0	25	≤0.03	S	≤0.03	S	≤0.03	S	≤0.12	S
2017–0795	Pn2	2017	Invasive	PT_0–0-0	0	28	≤0.03	S	0.008	S	≤0.03	S	0,03	S
2017–0758	Pn3	2017	Invasive	PT_0–0-0	0	27	≤0.03	S	≤0.03	S	≤0.03	S	≤0.12	S
2014–0010	Pn4	2014	Invasive	PT_0–0-0	0	30	≤0.03	S	≤0.03	S	≤0.03	S	≤0.12	S
2018-F8-32	Pn5	2018	Respiratory	PT_0–0-0	0	NA	≤0.03	S	≤0.03	S	≤0.03	S	≤0.12	S
2018-F1-175	Pn9	2018	Respiratory	PT_0–0-2	0	NA	≤0.03	S	≤0.03	S	≤0.03	S	≤0.12	S
2017–4068	Pn19	2017	Invasive	PT_0–0-3	0	28	≤0.03	S	0.008	S	≤0.03	S	0.06	S
2018-F5-219	Pn25	2018	Invasive	PT_1–0-0	0	NA	≤0.03	S	≤0.03	S	≤0.5	S	≤0.12	S
2018-F1-282	Pn26	2018	Respiratory	PT_12–0-0	0	NA	≤0.03	S	≤0.03	S	≤0.03	S	≤0.12	S
2010–0479	Pn28	2010	Respiratory	PT_13–14-26	0	NA	8	R	>4	R	4	R	>2	R
2010–0164	Pn29	2010	Respiratory	PT_15–12-18	0	NA	4	R	4	R	2	I	2	I
2003–0373	Pn34	2003	Invasive	PT_17–15-22	0	NA	0.5	I	0.5	I	0.25	S	0.25	S
2008–1108	Pn35	2008	Invasive	PT_17–15-22	0	NA	0.5	I	1	I	0.25	S	0.5	S
2004–1073	Pn41	2004	Invasive	PT_2–0-0	0	29	≤0.03	S	≤0.03	S	≤0.03	S	≤0.12	S
2010–0993	Pn42	2010	Invasive	PT_2–0-0	0	21	≤0.03	S	≤0.03	S	≤0.03	S	≤0.12	S
2005–0741	Pn43	2005	Invasive	PT_2–0-0	0	21	≤0.03	S	≤0.03	S	≤0.03	S	≤0.12	S
2006–0579	Pn44	2006	Invasive	PT_2–0-0	0	25	≤0.03	S	≤0.03	S	≤0.03	S	≤0.12	S
2010–0510	Pn45	2010	Invasive	PT_2–0-0	0	27	≤0.03	S	≤0.03	S	≤0.03	S	≤0.12	S
2002–1030	Pn46	2002	Invasive	PT_2–0-0	0	24	≤0.03	S	≤0.03	S	≤0.03	S	≤0.12	S
2012–0390	Pn47	2012	Invasive	PT_2–0-0	0	28	≤0.03	S	≤0.03	S	≤0.03	S	≤0.12	S
2006–1119	Pn48	2006	Invasive	PT_2–0-0	0	29	≤0.03	S	≤0.03	S	≤0.03	S	≤0.12	S
2007–1134	Pn49	2007	Invasive	PT_2–0-0	0	24	≤0.03	S	≤0.03	S	≤0.03	S	≤0.12	S
2009–1199	Pn50	2009	Invasive	PT_2–0-0	0	28	≤0.03	S	≤0.03	S	≤0.03	S	≤0.12	S
2010–0805	Pn51	2010	Invasive	PT_2–0-0	0	32	≤0.03	S	≤0.03	S	≤0.03	S	≤0.12	S
1999–0630	Pn52	1999	Invasive	PT_2–0-0	0	29	≤0.03	S	≤0.03	S	≤0.03	S	≤0.12	S
2005–0527	Pn53	2005	Invasive	PT_2–0-0	0	24	≤0.03	S	≤0.03	S	≤0.03	S	≤0.12	S
2015–0090	Pn54	2014	Invasive	PT_2–0-0	0	NA	≤0.03	S	0.016	S	≤0.03	S	0.016	S
2014–0403	Pn55	2014	Invasive	PT_2–0-0	0	29	≤0.03	S	≤0.03	S	≤0.03	S	≤0.12	S
2014–0054	Pn56	2014	Invasive	PT_2–0-0	0	27	≤0.03	S	≤0.03	S	≤0.03	S	≤0.12	S
2004–1186	Pn57	2004	Invasive	PT_2–0-0	0	NA	≤0.03	S	≤0.03	S	≤0.03	S	≤0.12	S
2007–0258	Pn58	2007	Invasive	PT_2–0-0	0	29	≤0.03	S	≤0.03	S	≤0.03	S	≤0.12	S
2013–0366	Pn59	2013	Invasive	PT_2–0-0	0	29	≤0.03	S	≤0.03	S	≤0.03	S	≤0.12	S
2016–0104	Pn60	2016	Invasive	PT_2–0-0	0	NA	≤0.03	S	0,016	S	≤0.03	S	≤0.12	S
2006–0194	Pn61	2006	Invasive	PT_2–0-0	0	NA	≤0.03	S	≤0.03	S	≤0.03	S	≤0.12	S
2014–0100	Pn62	2014	Invasive	PT_2–0-0	0	25	≤0.03	S	≤0.03	S	≤0.03	S	≤0.12	S
2012–0272	Pn63	2012	Invasive	PT_2–0-0	0	29	≤0.03	S	≤0.03	S	≤0.03	S	≤0.12	S
2009–0273	Pn64	2009	Invasive	PT_2–0-0	0	28	≤0.03	S	≤0.03	S	≤0.03	S	≤0.12	S
2013–0128	Pn65	2013	Invasive	PT_2–0-0	0	NA	≤0.03	S	0,03	S	≤0.03	S	≤0.12	S
2018-F1-70	Pn67	2018	Respiratory	PT_2–0-2	0	NA	≤0.03	S	0.06	S	≤0.03	S	≤0.12	S
2010–0976	Pn70	2010	Respiratory	PT_34–32-43	0	NA	2	I	2	I	≤0.5	S	1	I
2018-F11-21	Pn72	2018	Invasive	PT_3–6-5	0	NA	≤0.03	S	≤0.03	S	≤0.03	S	≤0.12	S
2018-F2-278	Pn73	2018	Invasive	PT_3–6-5	0	NA	≤0.03	S	≤0.03	S	≤0.03	S	≤0.12	S
2018-F11-42	Pn74	2018	Invasive	PT_3–6-5	0	NA	≤0.03	S	≤0.03	S	≤0.03	S	≤0.12	S
2006–0685	Pn75	2006	Respiratory	PT_4–7-7	0	NA	2	I	0,5	I	1	I	1	I
2018-F9-15	Pn77	2018	Invasive	PT_62–0-2	0	NA	≤0.03	S	≤0.03	S	≤0.03	S	≤0.12	S
2018-F7-86	Pn78	2018	Invasive	PT_7–1-1	0	NA	0.25	I	0.25	I	≤0.5	S	<0.12	S
2018-F2-190	Pn79	2018	Invasive	PT_7–1-1	0	NA	0.25	I	0.25	I	≤0.5	S	<0.12	S
2012–0263	Pn81	2012	Invasive	PT_78–0-0	0	28	≤0.03	S	≤0.03	S	≤0.03	S	≤0.12	S
2014–0149	Pn82	2014	Invasive	PT_78–0-0	0	28	≤0.03	S	≤0.03	S	≤0.03	S	≤0.12	S
2016–0201	Pn83	2016	Invasive	PT_78–0-0	0	28	≤0.03	S	≤0.03	S	≤0.03	S	≤0.12	S
2016–0041	Pn84	2016	Invasive	PT_78–0-0	0	29	≤0.03	S	≤0.03	S	≤0.03	S	≤0.12	S
2015–0120	Pn85	2015	Invasive	PT_78–0-0	0	NA	≤0.03	S	0.016	S	≤0.03	S	0.008	S
2015–0641	Pn86	2015	Invasive	PT_78–0-0	0	NA	≤0.03	S	0.016	S	≤0.03	S	0.008	S
2015–0096	Pn87	2015	Invasive	PT_78–0-0	0	NA	≤0.03	S	0.03	S	≤0–03	S	0.016	S
2007–0130	Pn88	2007	Respiratory	PT_8–67–103	0	NA	0.5	I	0.25	I	≤0.5	S	0.25	S

**Table 4:** Genotypic and phenotypic susceptibility in *Streptococcus pneumoniae* isolates with a new PBP-profile.

Isolate	ID	Year	Source of infection	Nearest PBP-profile	Substi-tutions	Oxacillin	Penicillin				Ceftriaxone			
						Pheno-typic mm	Geno-typic MIC	Geno-typic S-I-R	Pheno-typic MIC	Pheno-typic S-I-R	Geno-typic MIC	Geno-typic S-I-R	Pheno-typic MIC	Pheno-typic S-I-R
2003-24F	Pn6	1943	Respiratory	PT_0–0-0	1	NA	≤0.03	S	0.03	S	≤0.03	S	0.12	S
2018-F10-36	Pn7	2018	Other	PT_0–0-0	7	NA	≤0.03	S	≤0.03	S	≤0.03	S	≤0.12	S
2018-F8-14	Pn8	2018	Other	PT_0–0-0	7	NA	≤0.03	S	≤0.03	S	≤0.03	S	≤0.12	S
2018-F11-2	Pn10	2018	Respiratory	PT_0–0-28	8	NA	0.06	S	≤0.03	S	≤0.5	S	≤0.12	S
2017–0068	Pn11	2017	Invasive	PT_0–0-28	8	NA	0.06	S	≤0.03	S	≤0.5	S	≤0.12	S
2015–0223	Pn12	2015	Invasive	PT_0–0-28	8	NA	0.06	S	0.03	S	≤0.5	S	0.06	S
2017–0345	Pn13	2017	Invasive	PT_0–0-28	8	NA	0.06	S	≤0.03	S	≤0.5	S	≤0.12	S
2016–0350	Pn14	2016	Invasive	PT_0–0-28	8	NA	0.06	S	≤0.03	S	≤0.5	S	≤0.12	S
2007–0834	Pn15	2007	Invasive	PT_0–0-28	8	NA	0.06	S	≤0.03	S	≤0.5	S	≤0.12	S
2009–0811	Pn16	2009	Invasive	PT_0–0-28	8	NA	0.06	S	0.03	S	≤0.5	S	≤0.12	S
2014–0404	Pn17	2014	Invasive	PT_0–0-28	8	NA	0.06	S	≤0.03	S	≤0.5	S	≤0.12	S
2014–0747	Pn18	2014	Invasive	PT_0–0-28	8	NA	0.06	S	≤0.03	S	≤0.5	S	≤0.12	S
2016–0487	Pn20	2016	Invasive	PT_0–1-1	23	12	0.06	S	0.25	I	0.12	S	0.12	S
2013–0682	Pn21	2013	Invasive	PT_0–1-2	6	29	≤0.03	S	≤0.03	S	0.06	S	≤0.12	S
2014–0669	Pn22	2014	Invasive	PT_0–1-2	6	25	≤0.03	S	≤0.03	S	0.06	S	≤0.12	S
2015–0182	Pn23	2015	Invasive	PT_0–1-2	6	NA	≤0.03	S	0.016	S	0.06	S	0.016	S
2008–0266	Pn24	2008	Invasive	PT_0–1-2	6	24	≤0.03	S	≤0.03	S	0.06	S	≤0.12	S
2010–0812	Pn27	2010	Respiratory	PT_13–11-33	2	NA	4	R	>4	R	4	R	>2	R
2010–0661	Pn30	2010	Respiratory	PT_15–14-96	20	NA	8	R	4	R	>2	R	1	I
2010–129	Pn31	2010	Respiratory	PT_15–16-8	2	NA	1	I	2	I	1	I	1	I
2010–1247	Pn32	2010	Respiratory	PT_15–7-8	10	NA	4	R	4	R	1	I	2	I
2010–1060	Pn33	2010	Respiratory	PT_15–7-8	15	NA	4	R	4	R	1	I	2	I
2002–1038	Pn36	2002	Respiratory	PT_17–15-22	18	NA	0.5	I	0.5	I	0.25	S	0.50	S
2013–0699	Pn37	2013	Invasive	PT_17–15-22	26	NA	0.5	I	0.5	I	≤0.5	S	0.25	S
2015–0233	Pn38	2015	Invasive	PT_17–15-22	26	NA	0.5	I	0.5	I	0.25	S	0.25	S
2010–1006	Pn39	2010	Respiratory	PT_17–16-47	4	NA	2	I	2	I	2	I	1	I
2010–0484	Pn40	2010	Respiratory	PT_18–7-8	26	NA	4	R	2	I	1	I	2	I
2002–1043	Pn66	2002	Respiratory	PT_20–18-25	13	NA	1	I	0.12	I	≤0.5	S	0.25	S
2018-F8-24	Pn68	2018	Invasive	PT_2–6-2/ PT_1–6-0	2	NA	≤0.03	S	≤0.03	S	≤0.03	S	≤0.12	S
1999–1138	Pn69	1999	Respiratory	PT_27–11-8	20	NA	4	R	4	R	1	I	4	R
2010–1248	Pn71	2010	Respiratory	PT_34–89-147	3	NA	2	I	2	I	1	I	1	I
2018-F5-29	Pn76	2018	Respiratory	PT_54–61-92	43	NA	4	R	0.06	S	NA	NA	≤0.12	S
PROJ-57-2	Pn80	NA	Respiratory	PT_7–1-30	10	NA	0.25	I	0.12	I	≤0.5	S	0.25	S

All *S. pseudopneumoniae* isolates were phenotypic and genotypic susceptible toward penicillin and CA was 100%. Among *S. mitis* isolates of which 27 were phenotypic susceptible, CA was only 75.8% with six MiD, one MaD, and one VMaD. Among *S. oralis*, two isolates were phenotypically resistant toward penicillin; one was resistant and one was susceptible-increased-exposure by genotype. The remaining isolates were phenotypically susceptible, but all except one was susceptible-increased-exposure by genotypic prediction, resulting in a very poor CA of 8% (2 of 25) (Table 2). All phenotypic and genotypic MICs for penicillin are presented in Supplementary Tables S3, S5, S7 and S9. Compared to *S. pneumoniae*, the non-*S. pneumoniae* MGS had much poorer MIC correlation in Deming regression curves (Figures 1C–E).

### 3.4. Ceftriaxone: Correlation between genotypic MIC prediction and phenotypic susceptibility

For *S. pneumoniae* isolates with a recognized PBP-profile, 52 isolates were genotypic susceptible, but one isolate with a genotypic MIC of ≤0.5 μg/mL had a phenotypic MIC of 1 μg/mL resulting in one MiD (1.8%) and thus a CA of 98.2%. This was however a single dilution difference. For isolates with a new PBP-profile, one isolate had no available genotypic MIC in the CDC PBP-to-MIC database. CA for *S. pneumoniae* isolates was 93.8%, and two isolates had a MiD. PPV and NPV for non-susceptibility was 100 and 98%. *S. pseudopneumoniae* isolates with an available susceptibility had a CA of 100%. In *S. mitis*, 30 isolates were phenotypic susceptible toward ceftriaxone, and three isolates had reduced phenotypic susceptibility. CA was 86.2% with three MaD and one VMaD. In *S. oralis*, two isolates were both phenotypic and genotypic resistant toward ceftriaxone, and the remaining isolates were genotypic and phenotypic susceptible with a 100% CA (Table 2). Genotypic-phenotypic MIC correlation for ceftriaxone is shown in Figures 1F–J.

### 3.5. Genotypic prediction based on a single PBP subtype

We explored whether identifying the PBP subtype of only one of three PBPs in an isolate, could be enough to predict susceptibility (S, I or R), and this was the case for many subtypes. However, both susceptible and susceptible-increased-exposure isolates had common subtypes, such as PBP2b0, PBP2x0 or PBP2x2. For PBP1a only a few subtypes always predicted high level resistance, while other subtypes could be either I or R, or even S or R for one subtype (Supplementary Table S10). Therefore, it is necessary to identify the subtype of all three PBPs to predict susceptibility correctly.

### 3.6. Phylogenetic analysis of the PBP-TPD

**Figure 2:**
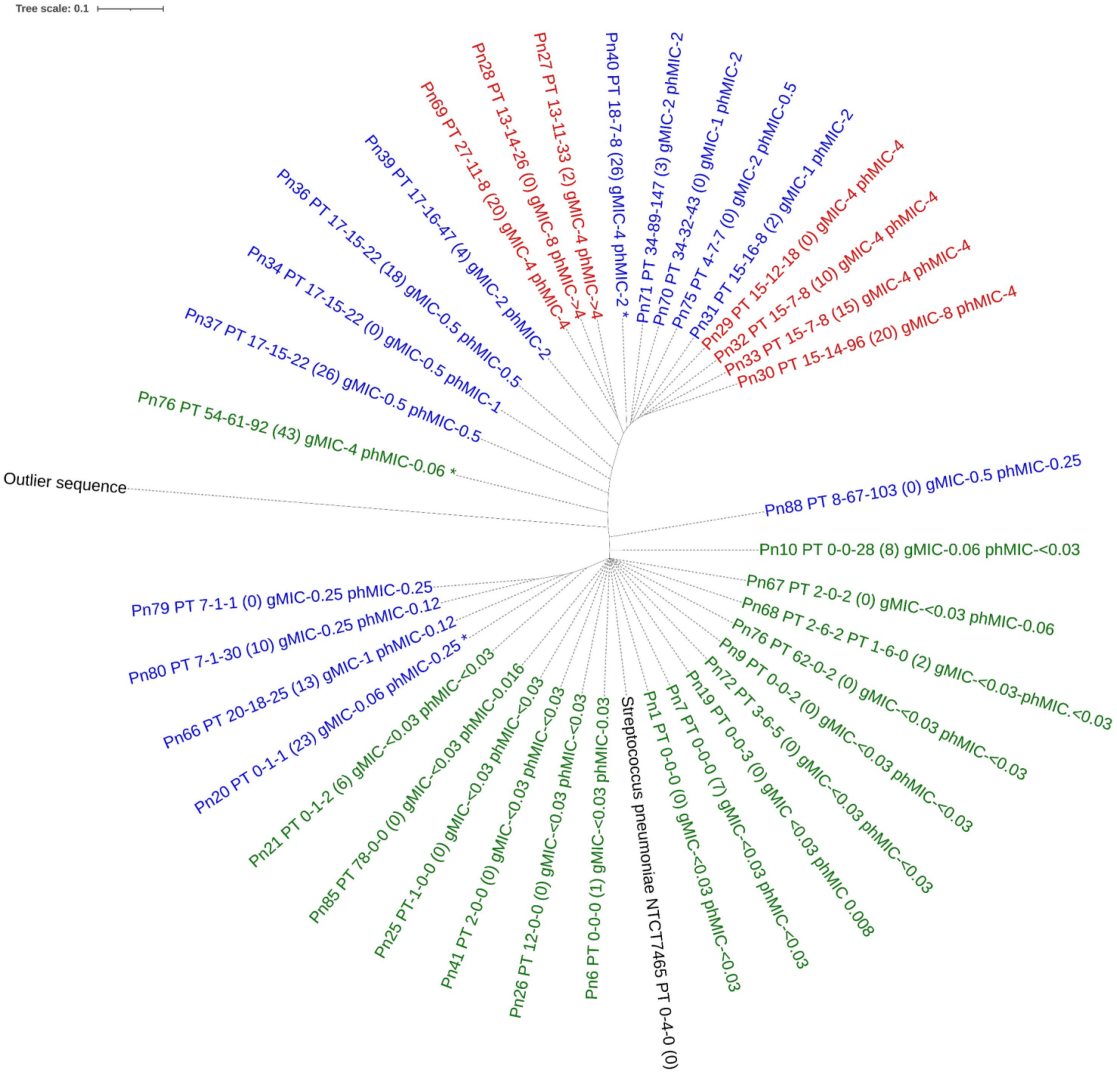
Phylogenetic tree of the concatenated amino acid sequence of PBP1a, PBP2b and PBP2x in *Streptococcus pneumoniae* with unique PBP-profiles. The tree was constructed using IQ-TREE software with a Blosum62 substitution model. Label contains isolate id, nearest PBP-profile (number of PBP amino acid substitutions), genotypic MIC, phenotypic MIC. Tree is colored by phenotypical susceptibility: Green is susceptible standard dosing regimen blue is susceptible increased exposure, red is resistant. The outlier sequence was constructed from *Streptococcus dysgoloctiae* AC2713. *indicates category disagreement.

**Figure 3:**
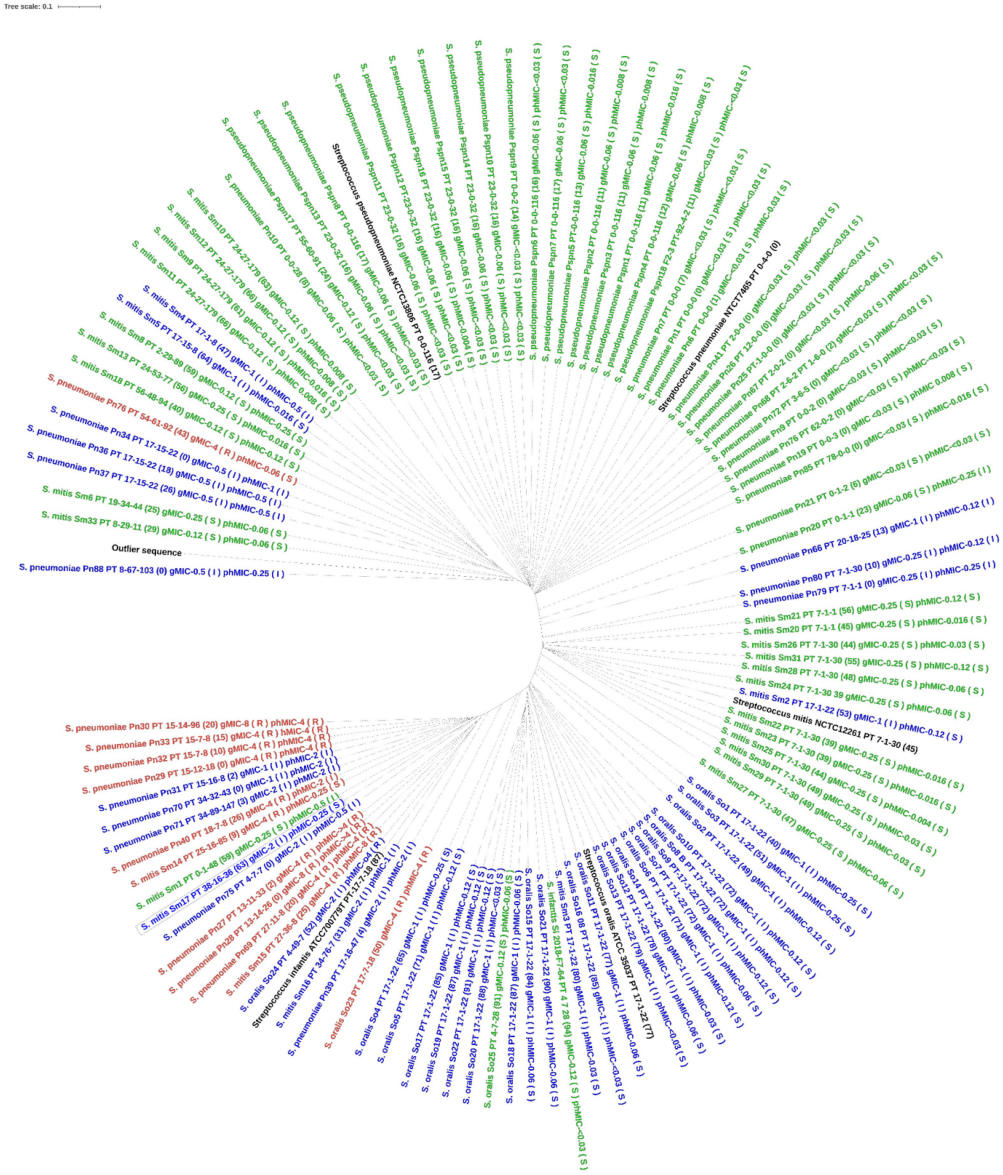
Phylogenetic tree of the concatenated PBP amino acid sequence in all mitis group streptococci. The tree was constructed using IQ-TREE software with Blosum62 substitution model. Label contains species and isolate id, nearest PBP-profile (number of PBP amino acid substitutions), genotypic MIC, phenotypic MIC. Tree is colored by genotypic susceptibility (green is susceptible, blue is susceptible increased exposure, red is resistant). The outlier sequence was constructed from *Streptococcus dysgalactiae* AC2713.

For *S. pneumoniae*, the concatenated PBP amino acid sequence of the susceptible isolates clustered with that of the *S. pneumoniae* reference genome (NTCT 7465). Phenotypic non-susceptible isolates clustered in one larger group including isolates being either susceptible-increased-exposure or resistant and in one smaller group, where one isolate, Pn20, was susceptible by genotype. This isolate had 23 substitutions to the nearest PBP-type, which may explain this finding. Only one unique profile (isolate, Pn76) was susceptible by phenotype, but resistant by genotype (MaD). Three isolates, all with PBP-profile PT_17–15-22, but with 0, 18, and 26 substitutions respectively, clustered together and had similar phenotypic MICs between 0.5 and 1 μg/mL, indicating no functional importance of these substitutions on susceptibility (Figure 2). When including all species in the analysis, the type strains for *S. pneumoniae, S. pseudopneumoniae, S. mitis* and *S. oralis* all clustered with susceptible isolates of that species. A separate cluster of susceptible *S. mitis* isolates was seen, and both *S. mitis* clusters had related non-susceptible *S. pneumoniae* isolates, suggesting a horizontal gene transfer between these two species. The one *S. infantis* isolate clustered with one *S. mitis* and one *S. oralis* isolate, both susceptible by phenotype, but susceptible-increased-exposure by genotype. A separate cluster included isolates of *S. pneumoniae, S. mitis* and *S. oralis* being susceptible-increased-exposure and resistant, and surprisingly, the *S. infantis* type strain was in this cluster (Figure 3).

## 4. Discussion

We studied genotypic prediction of β-lactam susceptibility in selected isolates of Danish *S. pneumoniae* and internationally collected non-*S. pneumoniae* MGS using a different method than previously described (Li et al., 2016; Metcalf et al., 2016b), namely BLAST analysis. This method only requires basic bioinformatic skills. Prediction was based on the concatenated amino acid sequence of the TPD of PBP1a, PBP2b and PBP2x in *S. pneumoniae*. There was an excellent prediction of β-lactam susceptibility in *S. pneumoniae* isolates with recognized PBP-profiles. There was a good prediction in *S. pneumoniae* isolates with new PBP-profiles when the nearest PBP-profile, identified by BLAST analysis, was used. Overall, the method performed well in Danish *S. pneumoniae* isolates. The method itself could also be applied to non-*S. pneumoniae* MGS, but none of these MGS species had a recognized PBP-profile. Using the database validated for *S. pneumoniae*, susceptibility to penicillin and ceftriaxone was poorly predicted for non-*S. pneumoniae* MGS. The method could potentially be used in non-*S. pneumoniae* MGS if a similar database is developed and validated using a larger collection of β-lactam susceptible and resistant isolates.

In our study, CA for *S. pneumoniae* was 100% for penicillin and 98.2% for ceftriaxone in isolates with a recognized PBP-profile. This is comparable with results from other studies. Li et al. (2016) tested three statistical models for genomic MIC prediction of β-lactam antibiotics. If a PBP-profile was present in the training dataset, EA was >98% and CA was >94%, for isolates in the test dataset [“mode MIC” (MM) statistical model]. In a subsequent study (Li et al., 2017), there was an acceptable performance for all β-lactams, with an EA of >97% and a CA of >90% (Random Forest model). Metcalf et al. (2016a) analyzed 2205 isolates with 145 recognized PBP-profiles and found that CA was 97.3%. Of 1724 isolates being phenotypic susceptible, 0.8% had wrong category predicted, this was 6% among 418 I-isolates, and 33.3% among 63 R- isolates.

We found that 54% of isolates had a new PBP-profile. Geographical differences in PBP-subtypes in circulating strains may explain this. It may also reflect that TPD-PBP substitutions continuously develop, due to the usage of β-lactam antibiotics which correlates to the proportion of non-susceptible *S. pneumoniae* isolates (Jensen et al., 2015). Regarding susceptibility prediction for isolates with a new PBP-profile, we found that CA for penicillin was 90.9%, MaD was 2.9% and there was no VMaD. Li et al. [14] found that EA and CA for penicillin was approximately 90% when the PBP-profile was not in the training dataset. MaD and VMaD were too high for all models in their study. Thus, regarding CA, our method, using a simple BLAST analysis, was not inferior to the previously published statistical models, both for isolates with a recognized and with a new PBP-profile.

Molecular based diagnostic tests are increasingly being explored as a supplement to culture, e.g., in bacterial meningitis (Moon et al., 2019; Nakagawa et al., 2019; Hong et al., 2020). This is particularly of value in pathogen positive, but culture negative specimens, e.g., due to prior antibiotic treatment. Genotypic susceptibility prediction could potentially ensure appropriate and narrow antibiotic treatment, when culture is not possible. In a clinical setting, a consensus is needed regarding the accepted CA, MaD and VMaD and whether this is different, depending on the type of infection. Using our BLAST method, genotypical susceptibility prediction may be safe to guide treatment, if a recognized PBP-profile is identified since CA was 100% for penicillin and 98.2% for ceftriaxone. In particular, when treating severe invasive infections we could have concerns using our method in isolates with new PBP-profiles with a CA of 90.9% (penicillin) and 93.8% (ceftriaxone). From a surveillance perspective, we find our results of the overall CA for *S. pneumoniae* acceptable, being 90.9–100%. Including culture negative specimens in the surveillance could provide more accurate estimates of susceptibility in circulating strains.

To our knowledge, this study is among the first published studies that explores phenotypic-genotypic susceptibility in non-*S. pneumoniae* MGS. For these species, and especially for the *S. mitis* species, there was a poor prediction of susceptibility using the database validated for *S. pneumoniae*. In *S. pneumoniae*, the number of substitutions in the PBPs correlates with reduced susceptibility (Li et al., 2016). For the non-*S. pneumoniae* MGS, both susceptible and non-susceptible isolates had many substitutions. Whether these substitutions reflect reduced susceptibility, or a different species is not clear. Although the prediction was poor, the method itself could easily be applied to the non-*S. pneumoniae* MGS, which had PBP-sequences of equal length as *S. pneumoniae*.

A limitation is that we studied a selected group of isolates. Our isolates were not representative of current clinical samples, except for isolates from the ODiD-project. Overall, there were more non-susceptible *S. pneumoniae* isolates than in the Danish national surveillance (DANMAP, 2019). Another limitation is that phenotypic testing was performed by different laboratories with different methods for susceptibility testing. Retesting was not possible in case of MIC discrepancies.

When using BLAST analysis the result is highly dependent on the number of isolates in the database. For *S. pneumoniae* our method provided an acceptable susceptibility prediction, but the robustness and reproducibility of our method needs to be tested in further studies using clinical isolates. In particular, validation for non-susceptible isolates is needed, since this has the greatest clinical impact. If databases for PBP-profile and phenotypic MIC correlation are improved, the need for validating the method for isolates with new PBP-profiles is less important.

We only included few non-*S. pneumoniae* MGS isolates. For these species, a much larger validation is needed using species-specific PBP-profile to MIC databases. This would be particularly challenging for the *S. mitis* species, since they have very diverse PBP-profiles, even among susceptible strains. This was also reflected in our data. A continuous surveillance of PBP-profiles and the correlating phenotypic susceptibility in circulating strains of *S. pneumoniae* and non-*S. pneumoniae* MGSis challenging but also necessary for the performance of the method.

In conclusion, in Danish *S. pneumoniae* isolates, our alternative method for prediction of β-lactam susceptibility from genomic data, using BLAST analysis, had a performance comparable to other studies for recognized PBP-profiles. Isolates with a new PBP-profile had an acceptable susceptibility prediction but the method needs further validation. The method could be applied to other MGS, but prediction was poor. The PBP classification system is an important step in the direction of genotypic susceptibility testing of streptococci for routine diagnostic purposes. This could improve susceptibility testing in pathogen positive, but culture negative clinical specimens.

## Data availability statement

Regarding access to the data, the datasets used and/or analyzed during the current study are available from the corresponding author on reasonable request.

## Author contributions

HE, KF, KS, and H-CS: conception of the work. HE, KF, AJ, CJ, XN, JC, PS, AR, FA, KS, H-CS, and the ODiD Consortium: laboratory investigation and bioinformatics, critical revision of the article, and final approval of the article. HE, KS, KF, and H-CS: drafting of the article. All authors contributed to the article and approved the submitted version.

## Conflict of interest

H-CS is involved with projects supported by Pfizer.

The remaining authors declare that the research was conducted in the absence of any commercial or financial relationships that could be construed as a potential conflict of interest.

## Publisher’s note

All claims expressed in this article are solely those of the authors and do not necessarily represent those of their affiliated organizations, or those of the publisher, the editors and the reviewers. Any product that may be evaluated in this article, or claim that may be made by its manufacturer, is not guaranteed or endorsed by the publisher.

## Footnotes

^1^ https://www.ncbi.nlm.nih.gov

^2^https://pathogen.watch version v3.11.9

^3^ https://eucast.org/

^4^ https://www.cdc.gov/streplab/pneumococcus/mic.html

^5^ https://www.ncbi.nlm.nih.gov/tools/gbench/

^6^ https://itol.embl.de/

## References

[ref1] Arbique J. C. Poyart C. Trieu-Cuot P. Quesne G. Carvalho M. D. G. S. Steigerwalt A. G.. (2004). Accuracy of phenotypic and genotypic testing for identification of *Streptococcus pneumoniae* and description of *Streptococcus pseudopneumoniae* sp. nov. J. Clin. Microbiol. 42, 4686–4696. doi: 10.1128/JCM.42.10.4686-4696.2004, PMID: 15472328 PMC522306

[ref2] Christensen J. S. Jensen T. G. Kolmos H. J. Pedersen C. Lassen A. (2012). Bacteremia with *Streptococcus pneumoniae*: Sepsis and other risk factors for 30-day mortality-a hospital-based cohort study. Eur. J. Clin. Microbiol. Infect. Dis. 31, 2719–2725. doi: 10.1007/s10096-012-1619-5, PMID: 22581362

[ref3] DANMAP (2009) “Appendix 2 Materials and Methods, Susceptibility Tesring,” in Use of antimicrobial agents and occurrence of antimicrobial resistance in bacteria from food animals, foods and humans in Denmark. eds. Jensen V. F. Hammerum A. M. (Statens Serum Institute; Danish Veterinary and Food Administration; Danish Medicines Agency; National Veterinary Institute, Technical University of Denmark and National Food Institute, Technical University of Denmark), 128–129.

[ref4] DANMAP (2019). “Resistance in human pathogens, 8.3.5 Streptococcus pneumoniae” in Use of antimicrobial agents and occurrence of antimicrobial resistance in bacteria from food animals, food and humans in Denmark. (Statens Serum Institut and National Food Institute, Technical University of Denmark), 126.

[ref5] Ellington M. J. Ekelund O. Aarestrup F. M. Canton R. Doumith M. Giske C.. (2017). The role of whole genome sequencing in antimicrobial susceptibility testing of bacteria: report from the EUCAST subcommittee. Clin. Microbiol. Infect. 23, 2–22. doi: 10.1016/j.cmi.2016.11.012, PMID: 27890457

[ref6] Epping L. van Tonder A. J. Gladstone R. A. Bentley S. D. Page A. J. Keane J. A. (2018). SeroBA: rapid high-throughput serotyping of *Streptococcus pneumoniae* from whole genome sequence data. Microb. Genomics 4, 1–6. doi: 10.1099/mgen.0.000186, PMID: 29870330 PMC6113868

[ref7] Fuursted K. Littauer P. J. Greve T. Scholz C. F. P. (2016). Septicemia with *Streptococcus pseudopneumoniae*: report of three cases with an apparent hepatic or bile duct association. Infect. Dis. 48, 636–639. doi: 10.3109/23744235.2016.1157896 27100044

[ref8] Habib G. Hoen B. Tornos P. Thuny F. Prendergast B. Vilacosta I.. (2009). Guidelines on the prevention, diagnosis, and treatment of infective endocarditis (new version 2009). Eur. Heart J. 30, 2369–2413. doi: 10.1093/eurheartj/ehp285, PMID: 19713420

[ref9] Hakenbeck R. Brückner R. Denapaite D. Maurer P. (2012). Molecular mechanisms of b -lactam resistance in. Future Microbiol. 7, 395–410. doi: 10.2217/fmb.12.2, PMID: 22393892

[ref10] Hong N. T. T. Nghia H. D. T. Thanh T. T. Lan N. P. H. Ny N. T. H. Ngoc N. M.. (2020). Cerebrospinal fluid MinION sequencing of 16S rRNA gene for rapid and accurate diagnosis of bacterial meningitis. J. Infect. 80, 469–496. doi: 10.1016/j.jinf.2019.12.011, PMID: 31891725 PMC7113840

[ref11] Jensen C. S. Iversen K. H. Dargis R. Shewmaker P. Rasmussen S. Christensen J. J.. (2021). *Streptococcus pseudopneumoniae*: use of whole-genome sequences to validate species identification methods. J. Clin. Microbiol. 59, 1–11. doi: 10.1128/JCM.02503-20, PMID: 33208473 PMC8111133

[ref12] Jensen A. Scholz C. F. P. Kilian M. (2016). Re-evaluation of the taxonomy of the mitis group of the genus *Streptococcus* based on whole genome phylogenetic analyses, and proposed reclassification of *Streptococcus dentisani* as *Streptococcus oralis* subsp. dentisani comb. nov., *Streptococcus tigurinus*. Int. J. Syst. Evol. Microbiol. 66, 4803–4820. doi: 10.1099/ijsem.0.001433, PMID: 27534397

[ref13] Jensen A. Valdórsson O. Frimodt-Møller N. Hollingshead S. Kilian M. (2015). Commensal streptococci serve as a reservoir for β-lactam resistance genes in *Streptococcus pneumoniae*. Antimicrob. Agents Chemother. 59, 3529–3540. doi: 10.1128/AAC.00429-15, PMID: 25845880 PMC4432199

[ref14] Jolley K. A. Bray J. E. Maiden M. C. J. (2018). Open-access bacterial population genomics: BIGSdb software, the PubMLST.org website and their applications [version 1; referees: 2 approved]. Wellcome Open Res. 3, 124–120. doi: 10.12688/wellcomeopenres.14826.1, PMID: 30345391 PMC6192448

[ref15] Kavalari I. D. Fuursted K. Krogfelt K. A. Slotved H. C. (2019). Molecular characterization and epidemiology of *Streptococcus pneumoniae* serotype 24F in Denmark. Sci. Rep. 9, 5481–5489. doi: 10.1038/s41598-019-41983-8, PMID: 30940899 PMC6445336

[ref16] Li Y. Metcalf B. J. Chochua S. Li Z. Gertz R. E. Walker H.. (2016). Penicillin-binding protein transpeptidase signatures for tracking and predicting β-lactam resistance levels in *Streptococcus pneumoniae*. mBio 7:e00756-16. doi: 10.1128/mBio.00756-16, PMID: 27302760 PMC4916381

[ref17] Li Y. Metcalf B. J. Chochua S. Li Z. Gertz R. E. Walker H.. (2017). Validation of β-lactam minimum inhibitory concentration predictions for pneumococcal isolates with newly encountered penicillin binding protein (PBP) sequences. BMC Genomics 18, 621–610. doi: 10.1186/s12864-017-4017-7, PMID: 28810827 PMC5558719

[ref18] Metcalf B. J. Chochua S. Gertz R. E. Li Z. Walker H. Tran T.. (2016a). Using whole genome sequencing to identify resistance determinants and predict antimicrobial resistance phenotypes for year 2015 invasive pneumococcal disease isolates recovered in the United States. Clin. Microbiol. Infect. 22, 1002.e1–1002.e8. doi: 10.1016/J.CMI.2016.08.001, PMID: 27542334

[ref19] Metcalf B. J. Gertz R. E. Gladstone R. A. Walker H. Sherwood L. K. Jackson D.. (2016b). Strain features and distributions in pneumococci from children with invasive disease before and after 13-valent conjugate vaccine implementation in the USA. Clin. Microbiol. Infect. 22, 60.e9–60.e29. doi: 10.1016/j.cmi.2015.08.027, PMID: 26363404 PMC4721534

[ref20] Moon J. Kim N. Kim T. J. Jun J. S. Lee H. S. Shin H. R.. (2019). Rapid diagnosis of bacterial meningitis by nanopore 16S amplicon sequencing: a pilot study. Int. J. Med. Microbiol. 309:151338. doi: 10.1016/j.ijmm.2019.151338, PMID: 31444101

[ref21] Nakagawa S. Inoue S. Kryukov K. Yamagishi J. Ohno A. Hayashida K.. (2019). Rapid sequencing-based diagnosis of infectious bacterial species from meningitis patients in Zambia. Clin. Transl. Immunol. 8:e01087-11. doi: 10.1002/cti2.1087, PMID: 31709051 PMC6831930

[ref22] Nguyen L. T. Schmidt H. A. Von Haeseler A. Minh B. Q. (2015). IQ-TREE: a fast and effective stochastic algorithm for estimating maximum-likelihood phylogenies. Mol. Biol. Evol. 32, 268–274. doi: 10.1093/molbev/msu300, PMID: 25371430 PMC4271533

[ref23] Rasmussen L. H. Dargis R. Højholt K. Christensen J. J. Skovgaard O. Justesen U. S.. (2016). Whole genome sequencing as a tool for phylogenetic analysis of clinical strains of Mitis group streptococci. Eur. J. Clin. Microbiol. Infect. Dis. 35, 1615–1625. doi: 10.1007/s10096-016-2700-2, PMID: 27325438

[ref24] Rebelo A. R. Ibfelt T. Bortolaia V. Leekitcharoenphon P. Hansen D. S. Nielsen H. L.. (2022). One Day in Denmark: Nationwide point-prevalence survey of human bacterial isolates and comparison of classical and whole-genome sequence-based species identification methods. PLoS One 17:e0261999-17. doi: 10.1371/journal.pone.0261999, PMID: 35148318 PMC8836320

[ref25] Smith A. M. Klugman K. P. (1998). Alterations in PBP 1a essential for high-level penicillin resistance in *Streptococcus pneumoniae*. Antimicrob. Agents Chemother. 42, 1329–1333. doi: 10.1128/AAC.42.6.1329, PMID: 9624469 PMC105597

[ref26] Van Der Linden M. Otten J. Bergmann C. Latorre C. Liñares J. Hakenbeck R. (2017). Insight into the diversity of penicillin-binding protein 2x alleles and mutations in viridans streptococci. Antimicrob. Agents Chemother. 61, 1–18. doi: 10.1128/AAC.02646-16, PMID: 28193649 PMC5404556

