# Supplementary material for: Predicting β-lactam susceptibility from the genome of Streptococcus pneumoniae and other mitis group streptococci
Source: Front Microbiol. 2023 Mar 2;14:1120023. doi: 10.3389/fmicb.2023.1120023 (PMC10018206; doi:10.3389/fmicb.2023.1120023)
Supplement: Supplementary file 1 [file Table_1.DOCX]

**Table S1: Description of geographical and anatomic origin of isolates included in the study**

|  | SSI^1^ | Aarhus^2^ | Slagelse/CDC^3^ | ODiD^4^ | Total |
| --- | --- | --- | --- | --- | --- |
| ***S. pneumoniae*** | 72 | 0 | 0 | 16 | 88 |
| *Anatomical site* |  |  |  |  |  |
| Blood or CSF^5^ | 54 |  |  | 8 | 62 |
| Respiratory | 18 |  |  | 6 | 24 |
| Other/NA |  |  |  | 2 | 2 |
| *Country:* Denmark | 72 |  |  | 16 | 88 |
| *Year* | 1999-2017^6^ |  |  | 2018 |  |
| ***S. pseudopneumoniae*** | 6 | 1 | 10 | 2 | 19 |
| *Anatomical site* |  |  |  |  |  |
| Blood or CSF | 4 |  |  |  | 4 |
| Respiratory |  | 1 | 10 | 2 | 13 |
| Other/NA | 2 |  |  |  | 2 |
| *Country* |  |  |  |  |  |
| Denmark | 6 | 1 |  | 2 | 9 |
| Canada |  |  | 10 |  | 10 |
| *Year* | 2003-2014 | NA | 2000-2002 | 2018 |  |
| ***S. mitis*** | 9 | 18 | 4 | 2 | 33 |
| *Anatomical site* |  |  |  |  |  |
| Blood or CSF | 2 | 8 | 4 |  | 14 |
| Respiratory | 2 | 10 |  | 2 | 14 |
| Other/NA | 5 |  |  |  | 5 |
| *Country* |  |  |  |  |  |
| Denmark | 9 | 9 | 4 | 2 | 24 |
| Sweden |  | 2 |  |  | 2 |
| Switzerland |  | 4 |  |  | 4 |
| UK |  | 1 |  |  | 1 |
| Japan |  | 1 |  |  | 1 |
| USA |  | 1 |  |  | 1 |
| *Year* | 1999-2003 | 1985-2004 | 2010-2014 | 2018 |  |
| ***S. oralis*** | 0 | 5 | 18 | 2 | 25 |
| *Anatomical site* |  |  |  |  |  |
| Blood or CSF |  | 2 | 18 | 1 | 21 |
| Respiratory |  | 3 |  |  | 3 |
| NA |  |  |  | 1 | 1 |
| *Country* |  |  |  |  |  |
| Denmark |  | 3 | 18 | 2 | 23 |
| Sweden |  | 1 |  |  | 1 |
| UK |  | 1 |  |  | 1 |
| *Year* |  | NA | 2010-2018 | 2018 |  |
| ***S. infantis*** |  |  |  | 1 | 1 |
| *Anatomical site:* Other |  |  |  | 1 | 1 |
| *Country:* Denmark |  |  |  | 1 | 1 |
| **Total** | 87 | 24 | 32 | 23 | 166 |

1) The Danish national Neisseria and Streptococcus Reference Laboratory (NSR), Statens Serum Institute (SSI)

2) The Department of Biomedicine, Faculty of Health, Aarhus University (Aarhus)

3) The Department of Clinical Microbiology, Slagelse Hospital (Slagelse) and Queen Elizabeth II Health Sciences Center, Halifax, Canada via the Centers for Disease Control and Prevention, Atlanta, GA, USA (CDC)

4) The One Day in Denmark project (ODiD)

5) Cerebrospinal fluid

6) One historical isolate from 1943 was also included
