# Supplementary material for: Predicting β-lactam susceptibility from the genome of Streptococcus pneumoniae and other mitis group streptococci
Source: Front Microbiol. 2023 Mar 2;14:1120023. doi: 10.3389/fmicb.2023.1120023 (PMC10018206; doi:10.3389/fmicb.2023.1120023)
Supplement: Supplementary file 2 [file Table_2.DOCX]

**Table S2: Unique PBP-profile and PBP1a-, PBP2b- and PBP2x-subtypes in *Streptococcus pseudopneumoniae***

|  | Number |  |  | PBP1a | | PBP2b | | PBP2x | |
| --- | --- | --- | --- | --- | --- | --- | --- | --- | --- |
| Nearest  PPB-profile | of  isolates | PBP profile identity % | Substitutions | Nearest subtype | Substitutions | Nearest subtype | Substitutions | Nearest subtype | Substitutions |
| Type strain  NCTC 13806  PT_0-0-116 | 1 | 98.1 | 17 | 1a55 | 5 | 2b29 | 3 | 2x149 | 4 |
| PT_0-0-116 | 3 | 98.8 | 11 | 1a92 | 6 | 2b0 | 0 | 2x116 | 4 |
| PT_0-0-116 | 1 | 98.7 | 12 | 1a55 | 5 | 2b0 | 0 | 2x116 | 6 |
| PT_0-0-116 | 1 | 98.6 | 13 | 1a55 | 5 | 2b60 | 1 | 2x116 | 4 |
| PT_0-0-116 | 1 | 98.3 | 16 | 1a55 | 5 | 2b29 | 2 | 2x116 | 6 |
| PT_0-0-116 | 1 | 98.1 | 17 | 1a55 | 5 | 2b29 | 2 | 2x116 | 7 |
| PT_0-0-116* | 1 | 98.1 | 17 | 1a55 | 5 | 2b29 | 3 | 2x149 | 4 |
| PT_0-0-2 | 1 | 98.5 | 14 | 1a55 | 5 | 2b29 | 2 | 2x2 | 4 |
| PT_23-0-32 | 1 | 98.3 | 16 | 1a86 | 2 | 2b29 | 2 | 2x149 | 4 |
| PT_23-0-32 | 6 | 98.3 | 16 | 1a55 | 5 | 2b29 | 2 | 2x149 | 4 |
| PT_55-60-91 | 1 | 97.4 | 24 | 1a55 | 5 | 2b60 | 1 | 2x179 | 7 |
| PT_92-4-2 | 1 | 98.8 | 11 | 1a92 | 6 | 2b0 | 0 | 2x34 | 3 |
| PT_94-121-201 | 1 | 97.5 | 20 | 1a94 | 6 | 2b29 | 2 | 2x116 | 4 |

*identical to type strain
