# Supplementary material for: Predicting β-lactam susceptibility from the genome of Streptococcus pneumoniae and other mitis group streptococci
Source: Front Microbiol. 2023 Mar 2;14:1120023. doi: 10.3389/fmicb.2023.1120023 (PMC10018206; doi:10.3389/fmicb.2023.1120023)
Supplement: Supplementary file 3 [file Table_3.DOCX]

**Table S3: Genotypic and phenotypic susceptibility in *Streptococcus pseudopneumoniae* isolates**

|  |  |  |  |  |  | Penicillin | | | | Ceftriaxone | | | |
| --- | --- | --- | --- | --- | --- | --- | --- | --- | --- | --- | --- | --- | --- |
| Isolate | ID | Year | Source of infection | Nearest  PBP-profile | Substitutions | Geno-typic  MIC | Geno-typic  S-I-R | Pheno-typic  MIC | Pheno-typic  S.I-R | Geno-typic  MIC | Geno-typic  S-I-R | Pheno-typic  MIC | Pheno-typic  S-I-R |
| 2011-0175 | Pspn1 | 2011 | Other | PT_0-0-116 | 11 | 0.06 | S | 0.016 | S | ≤0.5 | S | 0.06 | S |
| 2014-0338 | Pspn2 | 2014 | Invasive | PT_0-0-116 | 11 | 0.06 | S | 0.008 | S | ≤0.5 | S | 0.06 | S |
| 2014-61-14 | Pspn3 | 2014 | Invasive | PT_0-0-116 | 11 | 0.06 | S | 0.008 | S | ≤0.5 | S | 0.06 | S |
| 2003-0276 | Pspn4 | 2003 | Invasive | PT_0-0-116 | 12 | 0.06 | S | 0.008 | S | ≤0.5 | S | 0.06 | S |
| 2010-0141 | Pspn5 | 2010 | NA | PT_0-0-116 | 13 | 0.06 | S | 0.016 | S | ≤0.5 | S | 0.06 | S |
| PSPN_287-03 | Pspn6 | 2000-2 | Respiratory | PT_0-0-116 | 16 | 0.06 | S | ≤0.03 | S | ≤0.5 | S | ≤0.12 | S |
| PSPN_292-03 | Pspn7 | 2000-2 | Respiratory | PT_0-0-116 | 17 | 0.06 | S | ≤0.03 | S | ≤0.5 | S | ≤0.12 | S |
| PSPN_297-03 | Pspn8 | 2000-2 | Respiratory | PT_0-0-116 | 17 | 0.06 | S | ≤0.03 | S | ≤0.5 | S | ≤0.12 | S |
| PSPN_285-03 | Pspn9 | 2000-2 | Respiratory | PT_0-0-2 | 14 | ≤0.03 | S | ≤0.03 | S | ≤0.03 | S | ≤0.12 | S |
| 2018-F3-114 | Pspn10 | 2018 | Respiratory | PT_23-0-32 | 16 | 0.06 | S | ≤0.03 | S | NA | NA | ≤0.12 | S |
| PSPN_298-03 | Pspn11 | 2000-2 | Respiratory | PT_23-0-32 | 16 | 0.06 | S | ≤0.03 | S | NA | NA | ≤0.12 | S |
| PSPN_266-03 | Pspn12 | 2000-2 | Respiratory | PT_23-0-32 | 16 | 0.06 | S | ≤0.03 | S | NA | NA | ≤0.12 | S |
| PSPN_300-03 | Pspn13 | 2000-2 | Respiratory | PT_23-0-32 | 16 | 0.06 | S | ≤0.03 | S | NA | NA | ≤0.12 | S |
| PSPN_265-03 | Pspn14 | 2000-2 | Respiratory | PT_23-0-32 | 16 | 0.06 | S | ≤0.03 | S | NA | NA | ≤0.12 | S |
| PSPN_261-03 | Pspn15 | 2000-2 | Respiratory | PT_23-0-32 | 16 | 0.06 | S | ≤0.03 | S | NA | NA | ≤0.12 | S |
| SK674 | Pspn16 | NA | Respiratory | PT_23-0-32 | 16 | 0.06 | S | 0.004 | S | NA | NA | NA | NA |
| PSPN_269-03 | Pspn17 | 2000-2 | Respiratory | PT_55-60-91 | 24 | 0.12 | S | ≤0.03 | S | NA | NA | ≤0.12 | S |
| 2018-F2-3 | Pspn18 | 2018 | Respiratory | PT_92-4-2 | 11 | ≤0.03 | S | ≤0.03 | S | ≤0.03 | S | ≤0.12 | S |
| 2014-0565 | Pspn19 | 2014 | Invasive | PT_94-121-201 | 20 | 0.25 | S | 0.06 | S | 0.12 | S | 0.25 | S |
