# Supplementary material for: Predicting β-lactam susceptibility from the genome of Streptococcus pneumoniae and other mitis group streptococci
Source: Front Microbiol. 2023 Mar 2;14:1120023. doi: 10.3389/fmicb.2023.1120023 (PMC10018206; doi:10.3389/fmicb.2023.1120023)
Supplement: Supplementary file 4 [file Table_4.DOCX]

**Table S4: Unique PBP-profiles and PBP1a-, PBP2b- and PBP2x-subtypes in *Streptococcus mitis***

|  |  |  |  | PBP1a |  | PBP2b |  | PBP2x |  |
| --- | --- | --- | --- | --- | --- | --- | --- | --- | --- |
| Nearest  PPB-profile | Number  of isolates | PBP-profile identity % | Substitutions | Nearest subtype | Substitutions | Nearest subtype | Substitutions | Nearest subtype | Substitutions |
| Type stain  NCTC 12261  PT_7-1-30 | 1 | 95.08 | 45 | 1a27 | 10 | 2b1 | 4 | 2x165 | 4 |
| PT_0-1-48 | 1 | 93.54 | 59 | 1a38 | 31 | 2b1 | 4 | 2x109 | 1 |
| PT_17-1-22 | 1 | 94.19 | 53 | 1a27 | 7 | 2b1 | 6 | 2x165 | 18 |
| PT_17-1-22 | 1 | 91.24 | 80 | 1a17 | 19 | 2b1 | 16 | 2x28 | 16 |
| PT_17-15-8 | 1 | 94.85 | 47 | 1a43 | 30 | 2b15 | 4 | 2x20 | 2 |
| PT_17-15-8 | 1 | 92.99 | 64 | 1a43 | 30 | 2b15 | 5 | 2x40 | 13 |
| PT_19-34-11 | 1 | 97.3 | 25 | 1a94 | 0 | 2b15 | 4 | 2x98 | 7 |
| PT_2-29-89 | 1 | 94.75 | 47 | 1a24 | 33 | 2b48 | 0 | 2x29 | 0 |
| PT_2-29-89 | 1 | 93.54 | 59 | 1a24 | 33 | 2b15 | 4 | 2x29 | 1 |
| PT_24-27-179 | 1 | 93.33 | 61 | 1a4 | 27 | 2b52 | 20 | 2x179 | 5 |
| PT_24-27-179 | 1 | 93.11 | 63 | 1a4 | 27 | 2b15 | 18 | 2x179 | 5 |
| PT_24-27-179 | 1 | 92.78 | 66 | 1a4 | 32 | 2b52 | 20 | 2x179 | 5 |
| PT_24-27-179 | 1 | 92.78 | 66 | 1a4 | 27 | 2b15 | 8 | 2x179 | 8 |
| PT_24-53-77 | 1 | 93.87 | 56 | 1a4 | 33 | 2b15 | 5 | 2x79 | 6 |
| PT_25-16-85 | 1 | 99.01 | 9 | 1a25 | 7 | 2b16 | 0 | 2x8 | 0 |
| PT_27-36-8 | 1 | 97.26 | 25 | 1a96 | 12 | 2b76 | 0 | 2x43 | 6 |
| PT_34-76-7 | 1 | 96.61 | 31 | 1a18 | 26 | 2b76 | 99 | 2x100 | 1 |
| PT_38-16-36 | 1 | 93.10 | 63 | 1a18 | 34 | 2b16 | 97 | 2x127 | 17 |
| PT_56-48-94 | 1 | 95.52 | 40 | 1a43 | 23 | 2b60 | 2 | 2x94 | 0 |
| PT_6-0-167 | 1 | 94.97 | 45 | 1a43 | 14 | 2b48 | 0 | 2x29 | 2 |
| PT_7-1-1 | 1 | 95.19 | 44 | 1a60 | 10 | 2b18 | 7 | 2x1 | 14 |
| PT_7-1-1 | 1 | 93.87 | 56 | 1a60 | 13 | 2b18 | 14 | 2x93 | 13 |
| PT_7-1-30 | 1 | 95.73 | 39 | 1a27 | 12 | 2b1 | 4 | 2x165 | 6 |
| PT_7-1-30 | 1 | 95.73 | 39 | 1a27 | 12 | 2b1 | 6 | 2x165 | 5 |
| PT_7-1-30 | 1 | 95.73 | 39 | 1a27 | 22 | 2b18 | 9 | 2x165 | 9 |
| PT_7-1-30 | 1 | 95.19 | 44 | 1a10 | 2 | 2b1 | 7 | 2x165 | 10 |
| PT_7-1-30 | 1 | 95.19 | 44 | 1a7 | 12 | 2b1 | 9 | 2x165 | 7 |
| PT_7-1-30 | 1 | 94.86 | 47 | 1a27 | 14 | 2b1 | 5 | 2x104 | 19 |
| PT_7-1-30 | 1 | 94.75 | 48 | 1a10 | 3 | 2b1 | 5 | 2x165 | 17 |
| PT_7-1-30 | 1 | 94.64 | 49 | 1a27 | 12 | 2b1 | 8 | 2x158 | 16 |
| PT_7-1-30 | 1 | 94.64 | 49 | 1a27 | 12 | 2b1 | 13 | 2x165 | 13 |
| PT_7-1-30 | 1 | 93.98 | 55 | 1a10 | 2 | 2b1 | 21 | 2x107 | 21 |
| PT_75-0-77 | 1 | 94.22 | 50 | 1a17 | 17 | 2b60 | 5 | 2x117 | 10 |
| PT_8-29-11 | 1 | 96.8 | 29 | 1a94 | 0 | 2b15 | 4 | 2x67 | 9 |
