# Supplementary material for: Predicting β-lactam susceptibility from the genome of Streptococcus pneumoniae and other mitis group streptococci
Source: Front Microbiol. 2023 Mar 2;14:1120023. doi: 10.3389/fmicb.2023.1120023 (PMC10018206; doi:10.3389/fmicb.2023.1120023)
Supplement: Supplementary file 5 [file Table_5.DOCX]

**Table S5: Genotypic and phenotypic susceptibility in *Streptococcus mitis* isolates.**

|  |  |  |  |  |  | Penicillin | | | | Ceftriaxone | | | |
| --- | --- | --- | --- | --- | --- | --- | --- | --- | --- | --- | --- | --- | --- |
| Isolate | ID | Year | Source of infection | Nearest  PBP-profile | Substi-tutions | Geno-typic  MIC | Geno-typic  S-I-R | Pheno-typic  MIC | Pheno-typic  S.I-R | Geno-typic  MIC | Geno-typic  S-I-R | Pheno-typic  MIC | Pheno-typic  S-I-R |
| 2018-F3-96 | Sm1 | 2018 | Respiratory | PT_0-1-48 | 59 | 0.25 | S | 0.5 | I | 0.25 | S | 0.5 | S |
| SK637 | Sm2 | 1994 | Respiratory | PT_17-1-22 | 53 | 1 | I | 0.12 | S | 0.25 | S | 0.06 | S |
| 2003-24 | Sm3 | 2003 | NA | PT_17-1-22 | 80 | 1 | I | 0.03 | S | 0.25 | S | 0.12 | S |
| 14010803B | Sm4 | 2014 | Invasive | PT_17-15-8 | 47 | 1 | I | 0.5 | I | 1 | R | ≤0,12 | S |
| SK667 | Sm5 | 1994 | Invasive | PT_17-15-8 | 64 | 1 | I | 0.016 | S | 1 | R | 0.008 | S |
| SK1080 | Sm6 | NA | Invasive | PT_19-34-44 | 25 | 0.25 | S | 0.06 | S | 0.25 | S | 0.06 | S |
| 2010-172 | Sm7 | 2010 | Respiratory | PT_2-29-89 | 47 | 0.12 | S | 0.06 | S | NA | NA | 0.25 | S |
| 12021959B | Sm8 | 2012 | Invasive | PT_2-29-89 | 59 | 0.12 | S | 0.25 | S | NA | NA | 0.25 | S |
| SK569 | Sm9 | NA | Invasive | PT_24-27-179 | 61 | 0.12 | S | 0.016 | S | 0.06 | S | 0.008 | S |
| SK579 | Sm10 | NA | Invasive | PT_24-27-179 | 63 | 0.12 | S | 0.008 | S | 0.06 | S | 0.03 | S |
| SK616 | Sm11 | NA | Invasive | PT_24-27-179 | 66 | 0.12 | S | 0.008 | S | 0.06 | S | 0.004 | S |
| 2003-108 | Sm12 | 2003 | Invasive | PT_24-27-179 | 66 | 0.12 | S | 0.008 | S | 0.06 | S. | 0.06 | S |
| SK608 | Sm13 | 1994 | Respiratory | PT_24-53-77 | 56 | 0.25 | S | 0.016 | S | ≤0.5 | S | 0.016 | S |
| SK564 | Sm14 | NA | Respiratory | PT_25-16-85 | 9 | 4 | R | 0.25 | S | NA | NA | 0.25 | S |
| SK575 | Sm15 | NA | Invasive | PT_27-36-8 | 25 | 4 | R | 8 | R | 2 | R | 2 | R |
| SK578 | Sm16 | 1994 | Invasive | PT_34-76-7 | 31 | 2 | I | 1 | I | >2 | R | 2 | R |
| SK1126 | Sm17 | 2004 | Respiratory | PT_38-16-36 | 63 | 2 | I | 0.25 | S | >2 | R | 0.12 | S |
| SK597 | Sm18 | NA | Respiratory | PT_56-48-94 | 40 | 0.12 | S | 0.12 | S | NA | NA | 0.06 | S |
| 2018-F5-156 | Sm19 | 2018 | Respiratory | PT_6-0-167 | 45 | 0.25 | S | 0.5 | I | 0.5 | S | <0.12 | S |
| SK629 | Sm20 | 1994 | Respiratory | PT_7-1-1 | 44 | 0.25 | S | 0.016 | S | ≤0.5 | S | 0.016 | S |
| SK642 | Sm21 | 1994 | Respiratory | PT_7-1-1 | 56 | 0.25 | S | 0.12 | S | ≤0.5 | S | 0.06 | S |
| SK145 | Sm22 | NA | Respiratory | PT_7-1-30 | 39 | 0.25 | S | 0.016 | S | ≤0.5 | S | 0.008 | S |
| SK137 | Sm23 | 1985 | Respiratory | PT_7-1-30 | 39 | 0.25 | S | 0.016 | S | ≤0.5 | S | 0.03 | S |
| 2003-187 | Sm24 | 2003 | Other | PT_7-1-30 | 39 | 0.25 | S | 0.06 | S | ≤0.5 | S | 0.12 | S |
| SK1073 | Sm25 | NA | Invasive | PT_7-1-30 | 44 | 0.25 | S | 0004 | S | ≤0.5 | S | 0.008 | S |
| SK321 | Sm26 | NA | Respiratory | PT_7-1-30 | 44 | 0.25 | S | 0.03 | S | ≤0.5 | S | 0.016 | S |
| B-5756_13 | Sm27 | 2013 | Invasive | PT_7-1-30 | 47 | 0.25 | S | 0.06 | S | ≤0.5 | S | ≤0.12 | S |
| B-009152-10 | Sm28 | 2010 | Invasive | PT_7-1-30 | 48 | 0.25 | S | 0.06 | S | ≤0.5 | S | ≤0.12 | S |
| 2003-72 | Sm29 | 2003 | NA | PT_7-1-30 | 49 | 0.25 | S | 0.03 | S | ≤0.5 | S | 0.25 | S |
| 2003-19 | Sm30 | 2003 | NA | PT_7-1-30 | 49 | 0.25 | S | 0.03 | S | ≤0.5 | S | 0.5 | S |
| 2003-68 | Sm31 | 2003 | Invasive | PT_7-1-30 | 55 | 0.25 | S | 0.12 | S | ≤0.5 | S | 0.5 | S |
| 1999-86 | Sm32 | 1999 | Other | PT_75-0-77 | 50 | 0.06 | S | >4 | R | 0.06 | S | >2 | R |
| 2002-139 | Sm33 | 2002 | Respiratory | PT_8-29-11 | 29 | 0.12 | S | 0.06 | S | ≤0.5 | S | 0.25 | S |
