# Supplementary material for: Predicting β-lactam susceptibility from the genome of Streptococcus pneumoniae and other mitis group streptococci
Source: Front Microbiol. 2023 Mar 2;14:1120023. doi: 10.3389/fmicb.2023.1120023 (PMC10018206; doi:10.3389/fmicb.2023.1120023)
Supplement: Supplementary file 6 [file Table_6.DOCX]

**Table S6: Unique PBP-profile and PBP1a-, PBP2b- and PBP2x-subtypes in *Streptococcus oralis***

|  | Number |  |  | PBP1a | | PBP2b |  | PBP2x |  |
| --- | --- | --- | --- | --- | --- | --- | --- | --- | --- |
| Nearest  PPB-profile | of  isolates | PBP-profile identity % | Substitutions | Nearest subtype | Substitutions | Nearest subtype | Substitutions | Nearest  subtype | Substitutions |
| NCBI reference  ATCC 35037  PT_17-1-22 | 1 | 91.57 | 77 | 1a17 | 5 | 2b37 | 34 | 2x111 | 11 |
| PT_17-1-22 | 1 | 95.62 | 40 | 1a17 | 5 | 2b18 | 4 | 2x111 | 7 |
| PT_17-1-22 | 1 | 94.63 | 49 | 1a17 | 6 | 2b18/2b103 | 13 | 2x111 | 18 |
| PT_17-1-22 | 1 | 94.41 | 51 | 1a17 | 5 | 2b18 | 15 | 2x111 | 18 |
| PT_17-1-22 | 1 | 92.88 | 65 | 1a17 | 5 | 2b99 | 33 | 2x111 | 16 |
| PT_17-1-22 | 1 | 92.22 | 71 | 1a17 | 5 | 2b37 | 34 | 2x99 | 17 |
| PT_17-1-22 | 1 | 92.22 | 71 | 1a17 | 5 | 2b41 | 34 | 2x111 | 7 |
| PT_17-1-22 | 3 | 92.11 | 72 | 1a17 | 5 | 2b37 | 35 | 2x111 | 16 |
| PT_17-1-22 | 1 | 92.11 | 72 | 1a17 | 6 | 2b37 | 34 | 2x111 | 18 |
| PT_17-1-22 | 1 | 91.57 | 77 | 1a17 | 5 | 2b37 | 34 | 2x111 | 11 |
| PT_17-1-22 | 1 | 91.46 | 78 | 1a17 | 5 | 2b37 | 35 | 2x111 | 11 |
| PT_17-1-22 | 1 | 91.35 | 79 | 1a17 | 6 | 2b37 | 35 | 2x111 | 11 |
| PT_17-1-22 | 1 | 91.24 | 80 | 1a17 | 5 | 2b37 | 35 | 2x111 | 13 |
| PT_17-1-22 | 1 | 90.9 | 84 | 1a17 | 14 | 2b18/2b103 | 33 | 2x111 | 12 |
| PT_17-1-22 | 1 | 90.69 | 85 | 1a17 | 16 | 2b18/2b103 | 34 | 2x111 | 11 |
| PT_17-1-22 | 1 | 90.69 | 85 | 1a17 | 20 | 2b18/2b103 | 34 | 2x111 | 18 |
| PT_17-1-22 | 1 | 90.47 | 87 | 1a17 | 18 | 2b1 | 35 | 2x111 | 11 |
| PT_17-1-22 | 1 | 90.47 | 87 | 1a17 | 20 | 2b18/2b1 | 33 | 2x111 | 15 |
| PT_17-1-22 | 1 | 90.36 | 88 | 1a17 | 19 | 2b1 | 35 | 2x111 | 10 |
| PT_17-1-22 | 1 | 90.14 | 90 | 1a17 | 25 | 2b18 | 33 | 2x111 | 5 |
| PT_17-1-22 | 1 | 90.03 | 91 | 1a17 | 22 | 2b18/2b103 | 34 | 2x111 | 12 |
| PT_17-7-18 | 1 | 94.53 | 50 | 1a17 | 9 | 2b37 | 23 | 2x100 | 2 |
| PT_4-49-7 | 1 | 94.31 | 52 | 1a17 | 41 | 2b49 | 1 | 2x55 | 3 |
| PT_4-7_28 | 1 | 90.04 | 91 | 1a17 | 21 | 2b18/2b103 | 33 | 2x111 | 11 |
