# Supplementary material for: Predicting β-lactam susceptibility from the genome of Streptococcus pneumoniae and other mitis group streptococci
Source: Front Microbiol. 2023 Mar 2;14:1120023. doi: 10.3389/fmicb.2023.1120023 (PMC10018206; doi:10.3389/fmicb.2023.1120023)
Supplement: Supplementary file 7 [file Table_7.DOCX]

**Table S7: Genotypic and phenotypic susceptibility in *Streptococcus oralis* isolates.**

|  |  |  |  |  |  | Penicillin | | | | Ceftriaxone | | | |
| --- | --- | --- | --- | --- | --- | --- | --- | --- | --- | --- | --- | --- | --- |
| Isolate | ID | Year | Source of infection | Nearest  PBP-profile | Substi-tutions | Geno-typic  MIC | Geno-typic  S-I-R | Pheno-typic  MIC | Pheno-typic  S.I-R | Geno-typic  MIC | Geno-typic  S-I-R | Pheno-typic  MIC | Pheno-typic  S-I-R |
| B603216-18 | So1 | 2018 | Invasive | PT_17-1-22 | 40 | 1 | I | 0.25 | S | 0.25 | S | 0.25 | S |
| 14030322B | So2 | 2014 | Invasive | PT_17-1-22 | 49 | 1 | I | 0.12 | S | 0.25 | S | ≤0.12 | S |
| 2018-F5-197 | So3 | 2018 | Invasive | PT_17-1-22 | 51 | 1 | I | 0.25 | S | 0.25 | S | ≤0.12 | S |
| SK610 | So4 | NA | Respiratory | PT_17-1-22 | 65 | 1 | I | 0.25 | S | 0.25 | S | 0.5 | S |
| 18514620B | So5 | 2018 | Invasive | PT_17-1-22 | 71 | 1 | I | 0.12 | S | 0.25 | S | 0.5 | S |
| 16011541B | So6 | 2016 | Invasive | PT_17-1-22 | 71 | 1 | I | 0.12 | S | 0.25 | S | ≤0.12 | S |
| B_19836_11 | So7 | 2011 | Invasive | PT_17-1-22 | 72 | 1 | I | 0.06 | S | 0.25 | S | ≤0.12 | S |
| B_007274_11 | So8 | 2011 | Invasive | PT_17-1-22 | 72 | 1 | I | 0.12 | S | 0.25 | S | ≤0.12 | S |
| Y_11577_11 | So9 | 2011 | Invasive | PT_17-1-22 | 72 | 1 | I | 0.12 | S | 0.25 | S | ≤0.12 | S |
| 2018-F7-87 | So10 | 2018 | NA | PT_17-1-22 | 72 | 1 | I | 0.12 | S | 0.25 | S | ≤0.12 | S |
| 14015779B | So11 | 2014 | Invasive | PT_17-1-22 | 77 | 1 | I | 0.06 | S | 0.25 | S | ≤0.12 | S |
| SK10 | So12 | NA | Invasive | PT_17-1-22 | 78 | 1 | I | 0.06 | S | 0.25 | S | 0.06 | S |
| 14010395B | So13 | 2014 | Invasive | PT_17-1-22 | 79 | 1 | I | ≤0.03 | S | 0.25 | S | ≤0.12 | S |
| SK100 | So14 | NA | Respiratory | PT_17-1-22 | 80 | 1 | I | 0.03 | S | 0.25 | S | 0.03 | S |
| B576280-18 | So15 | 2018 | Invasive | PT_17-1-22 | 84 | 1 | I | 0.06 | S | 0.25 | S | ≤0.12 | S |
| Y-052157-08 | So16 | 2008 | Invasive | PT_17-1-22 | 85 | 1 | I | ≤0.03 | S | 0.25 | S | ≤0.12 | S |
| 16010772B | So17 | 2016 | Invasive | PT_17-1-22 | 85 | 1 | I | 0.12 | S | 0.25 | S | ≤0.12 | S |
| SK1074 | So18 | NA | Invasive | PT_17-1-22 | 87 | 1 | I | 0.06 | S | 0.25 | S | 0.06 | S |
| 16012229B | So19 | 2016 | Invasive | PT_17-1-22 | 87 | 1 | I | 0.12 | S | 0.25 | S | 0.25 | S |
| Æ01011289 | So20 | NA | Invasive | PT_17-1-22 | 88 | 1 | I | ≤0.03 | S | 0.25 | S | ≤0.12 | S |
| Y-5914_11 | So21 | 2011 | Invasive | PT_17-1-22 | 90 | 1 | I | 0.06 | S | 0.25 | S | 0.25 | S |
| B-003802-10 | So22 | 2010 | Invasive | PT_17-1-22 | 91 | 1 | I | 0.12 | S | 0.25 | S | ≤0.12 | S |
| 14031600Y | So23 | 2014 | Invasive | PT_17-7-18 | 50 | 4 | R | 4 | R | 2 | R | >2 | R |
| 18022363Y | So24 | 2018 | Invasive | PT_4-49-7 | 52 | 2 | I | >4 | R | 1 | R | >2 | R |
| SK304 | So25 | NA | Respiratory | PT_4-7_28 | 91 | 0.12 | S | 0.06 | S | 0.06 | S | 0.03 | S |
