# Supplementary material for: Predicting β-lactam susceptibility from the genome of Streptococcus pneumoniae and other mitis group streptococci
Source: Front Microbiol. 2023 Mar 2;14:1120023. doi: 10.3389/fmicb.2023.1120023 (PMC10018206; doi:10.3389/fmicb.2023.1120023)
Supplement: Supplementary file 8 [file Table_8.docx]

**Table S8: Unique PBP-types and PBP1a-, PBP2b- and PBP2x-subtypes in *Streptococcus infantis***

|  |  |  |  | PBP1a | | PBP2b |  | PBP2x |  |
| --- | --- | --- | --- | --- | --- | --- | --- | --- | --- |
| Nearest  PPB-profile | Number  of  isolates | PBP-profile identity % | Substitutions | Nearest subtype | Substitutions | Nearest subtype | Substitutions | Nearest subtype | Substitutions |
| NCBI reference  ACTT 700779T  PT_17_7_18 | 1 | 90.48 | 87 | 1a17 | 36 | 2b1/2b103 | 31 | 2x189 | 7 |
| PT_4-7-28 | 1 | 89.72 | 94 | 1a4 | 35 | 2b1 | 30 | 2x28 | 24 |
