# Supplementary material for: Predicting β-lactam susceptibility from the genome of Streptococcus pneumoniae and other mitis group streptococci
Source: Front Microbiol. 2023 Mar 2;14:1120023. doi: 10.3389/fmicb.2023.1120023 (PMC10018206; doi:10.3389/fmicb.2023.1120023)
Supplement: Supplementary file 9 [file Table_9.docx]

**Table S9: Genotypic and phenotypic susceptibility in a *Streptococcus infantis* isolate.**

|  |  |  |  |  |  | Penicillin | | | | Ceftriaxone | | | |
| --- | --- | --- | --- | --- | --- | --- | --- | --- | --- | --- | --- | --- | --- |
| Isolate | ID | Year | Source of infection | Nearest  PBP-profile | Substi-tutions | Geno-typic  MIC | Geno-typic  S-I-R | Pheno-typic  MIC | Pheno-typic  S.I-R | Geno-typic  MIC | Geno-typic  S-I-R | Pheno-typic  MIC | Pheno-typic  S-I-R |
| 2018-F7-64 | Si1 | 2018 | Other | PT_4-7-28 | 94 | 0.12 | S | ≤0.03 | S | 0.06 | S | ≤0.12 | S |
