# Supplementary material for: Predicting β-lactam susceptibility from the genome of Streptococcus pneumoniae and other mitis group streptococci
Source: Front Microbiol. 2023 Mar 2;14:1120023. doi: 10.3389/fmicb.2023.1120023 (PMC10018206; doi:10.3389/fmicb.2023.1120023)
Supplement: Supplementary file 10 [file Table_10.docx]

**Table S10: PBP-subtypes and their correlation with susceptibility.** In brackets total number of isolates with that subtype – number of PBP-profiles including this subtype

| S-I-R | **PBP1a** | **PBP2b** | **PBP2x** |
| --- | --- | --- | --- |
| S | PBP1a3(110-6), PBP1a5(1-1), PBP1a9(47-2), PBP1a11(49-5), PBP1a14(2-1), PBP1a22(1-1), PBP1a30(2-1), PBP1a37(1-1), PBP1a44(1-1), PBP1a49(1-1), PBP1a50(3-1), PBP1a62(3-1), PBP1a63(1-1), PBP1a64(1-1), PBP1a68(1-1), PBP1a69(1-1), PBP1a70(1-1), PBP1a72(1-1), PBP1a73(1-1), PBP1a76(6-1), PBP1a77(2-1), PBP1a78(3-1), PBP1a89(1-1), PBP1a91(4-1), PBP1a92(1-1), PBP1a93(1-1), PBP1a99(1-1), PBP1a100(1-1), PBP1a101(1-1) | PBP2b2(408-22), PBP2b3(182-4), PBP2b4(358-35), PBP2b5(91-3), PBP2b10(34-6), PBP2b44(13-3), PBP2b45(1-1), PBP2b55(1-1), PBP2b59(1-1), PBP2b69(1-1), PBP2b70(1-1), PBP2b71(1-1),  PBP2b72(1-1), PBP2b78(3-1), PBP2b79(1-1), PBP2b80(1-1), PBP2b81(1-1), PBP2b82(1-1), PBP2b88(1-1), PBP2b100(1-1), PBP2b102(1-1), PBP2b104(1-1), PBP2b105(1-1), PBP2b106(1-1), PBP2b107(1-1), PBP2b108(1-1), PBP2b119(1-1) | PBP2x3(223-14), PBP2x4(12-1), PBP2x5(111-5), PBP2x6(243-13), PBP2x10(29-4), PBP2x12(12-3), PBP2x14(11-1), PBP2x15(1-1), PBP2x17(30-2), PBP2x21(3-2), PBP2x23(6-3), PBP2x27(5-1), PBP2x29(1-1), PBP2x31(1-1), PBP2x32(2-1), PBP2x34(23-2), PBP2x38(1-1), PBP2x39(1-1), PBP2x41(2-1), PBP2x42(1-1), PBP2x49(2-1), PBP2x53(7-4), PBP2x54(4-1), PBP2x59(19-2), PBP2x60(1-1), PBP2x61(1-1), PBP2x64(9-1), PBP2x66(3-3), PBP2x69(3-3), PBP2x71(2-1), PBP2x74(1-1), PBP2x75(1-1), PBP2x78(3-1), PBP2x81(1-1), PBP2x83(2-1), PBP2x84(2-2), PBP2x88(1-1), PBP2x93(1-1), PBP2x95(1-1), PBP2x99(1-1), PBP2x101(1-1), PBP2x110(1-1), PBP2x111(13-1), PBP2x116(1-1), PBP2x119(1-1), PBP2x120(9-1), PBP2x123(1-1), PBP2x124(4-1), PBP2x125(1-1), PBP2x128(1-1), PBP2x132(1-1), PBP2x134(1-1), PBP2x136(1-1), PBP2x137(2-1), PBP2x139(1-1), PBP2x145(1-1), PBP2x148(1-1), PBP2x153(2-2), PBP2x165(1-1), PBP2x166(1-1), PBP2x168(1-1), PBP2x170(1-1), PBP2x172(6-1), PBP2x174(1-1), PBP2x175(1-1), PBP2x176(1-1), PBP2x178(2-1), PBP2x180(2-1), PBP2x191(1-1), PBP2x192(1-1), PBP2x193(1-1), PBP2x195(1-1), PBP2x196(1-1), PBP2x197(1-1), PBP2x199(1-1) |
| S / I | PBP1a0(684-46/194-11), PBP1a1(667-34/4-4), PBP1a2(1209-54/15-9), PBP1a8(1-1/41-9),  PBP1a12(103-14/2-2), PBP1a19(1-1/43-7), PBP1a21(1-1/1-1), PBP1a23(147-24/3-2), PBP1a24(2-2/35-16) | PBP2b0(1464-93/35-7), PBP2b1(2-1/178-13), PBP2b6(140-13/2-1), PBP2b20(1-1/1-1), PBP2b23(2-1/1-1), PBP2b26(56-8/1-1),  PBP2b27(3-3/-66-16), PBP2b34(1-1/2-1), PBP2b40(4-1/1-1) | PBP2x0(821-35/1-1), PBP2x1(2-1/42-7), PBP2x2(1369-43/1-1), PBP2x9(1-1/28-2), PBP2x11(10-5/93-10), PBP2x13(1-1/7-2), PBP2x24(1-1/27-2), PBP2x28(1-1/42-4), PBP2x44(2-2/4-3), PBP2x50(7-1/4-1), PBP2x57(1-1/1-1), PBP2x77(1-1/4-3) |
| I | PBP1a4(130-15), PBP1a6(10-5), PBP1a7(33-5), PBP1a10(2-1), PBP1a16(2-1), PBP1a20(2-2), PBP1a31(1-1), PBP1a33(4-1), PBP1a34(9-6), PBP1a35(1-1), PBP1a36(2-1), PBP1a40(1-1), PBP1a42(5-1), PBP1a43(1-1), PBP1a46(1-1), PBP1a47(1-1), PBP1a51(1-1), PBP1a55(1-1), PBP1a56(1-1), PBP1a58(1-1), PBP1a60(1-1), PBP1a66(1-1), PBP1a67(1-1), PBP1a71(2-1), PBP1a74(1-1), PBP1a79(2-2), PBP1a80(1-1), PBP1a90(1-1), PBP1a94(2-2), PBP1a97(1-1), PBP1a98(1-1), PBP1a101(1-1) | PBP2b8(27-1), PBP2b13(2-1), PBP2b18(2-2), PBP2b19(1-1), PBP2b21(1-1), PBP2b22(1-1), PBP2b25(2-2), PBP2b29(61-8), PBP2b30(16-8), PBP2b32(1-1), PBP2b33(3-2), PBP2b34(1-1), PBP2b35(1-1), PBP2b37(2-2), PBP2b39(6-3), PBP2b41(2-1), PBP2b42(5-1), PBP2b43(1-1), PBP2b46(2-1), PBP2b50(1-1), PBP2b52(1-1), PBP2b53(4-3), PBP2b56(1-1), PBP2b57(2-1), PBP2b58(1-1), PBP2b60(1-1), PBP2b65(1-1), PBP2b66(1-1), PBP2b67(1-1), PBP2b68(1-1), PBP2b73(1-1), PBP2b74(1-1), PBP2b76(1-1), PBP2b85(1-1), PBP2b86(1-1), PBP2b87(1-1), PBP2b89(2-1), PBP2b101(1-1), PBP2b103(1-1), PBP2b110(1-1), PBP2b120(1-1) | PBP2x19(2-1), PBP2x20(2-2), PBP2x22(8-2), PBP2x30(1-1), PBP2x40(2-1), PBP2x43(1-1), PBP2x46(3-3), PBP2x48(4-1), PBP2x51(1-1), PBP2x56(5-3), PBP2x65(1-1), PBP2x72(2-2), PBP2x73(4-2), PBP2x76(1-1), PBP2x77(1-1), PBP2x79(1-1), PBP2x80(2-2), PBP2x86(2-1), PBP2x89(1-1), PBP2x91(2-2), PBP2x94(1-1), PBP2x97(1-1), PBP2x100(1-1), PBP2x103(1-1), PBP2x104(1-1), PBP2x109(1-1), PBP2x112(1-1), PBP2x114(2-2), PBP2x117(1-1), PBP2x127(1-1), PBP2x129(2-2), PBP2x130(1-1), PBP2x131(2-1), PBP2x133(1-1), PBP2x135(1-1), PBP2x138(1-1), PBP2x140(1-1), PBP2x141(2-1), PBP2x142(1-1), PBP2x143(3-1), PBP2x146(1-1), PBP2x147(2-1), PBP2x167(1-1), PBP2x169(1-1), PBP2x171(1-1), PBP2x173(1-1), PBP2x177(1-1), PBP2x179(1-1), PBP2x181(1-1), PBP2x194(1-1), PBP2x198(2-1), PBP2x200(1-1), PBP2x201(1-1), PBP2x203(1-1) |
| S / R | PBP1a38(2-1/2-1), PBP1a45(1-1/2-1), PBP1a75(1-1/1-1) |  |  |
| S / I / R |  | PBP2b12(2-2/15-6/36-6), PBP2b16 (2-1/12-8/4-3) | PBP2x36(2-1/7-6/3-3) |
| I / R | PBP1a13(9-7/131/14), PBP1a 15(6-4/33-6), PBP1a 17(15-6/2-2), PBP1a18(2-2/6-4), PBP1a 25(3-3/3-3), PBP1a 27(4-4/32-7), PBP1a39(3-3/1-1), PBP1a41(1-1/1-1) | PBP2b7(124-11/3-3), PBP2b11(2-2/112-10), PBP2b15(6-1/1-1), PBP2b36(3-3/31-8), PBP2b38(4-4/3-1), PBP2b49(1-1/1-1), PBP2b62(2-2/1-1) | PBP2x7(126-10/1-1), PBP2x8(69-24/68-16), PBP2x16(1-1/108-4), PBP2x18(1-1/32-4), PBP2x20(1-1/5-1), PBP2x33(1-1/4-2), PBP2x47(3-2/1-1), PBP2x52(2-2/5-3), PBP2x55(1-1/1-1) |
| R | PBP1a28(1-1), PBP1a29(2-1), PBP1a31(2-1), PBP1a48(1-1), PBP1a52(1-1), PBP1a54(1-1), PBP1a57(1-1) | PBP2b14(21-6), PBP2b17(1-1), PBP2b28(1-1), PBP2b51(1-1), PBP2b54(1-1), PBP2b61(1-1), PBP2b63(1-1), PBP2b77(1-1), PBP2b83(3-1) | PBP2x26(13-4), PBP2x37(1-1), PBP2x62(1-1), PBP2x63(2-1), PBP2x70(1-1), PBP2x82(1-1), PBP2x85(1-1), PBP2x92(1-1), PBP2x96(2-2), PBP2x113(1-1), PBP2x115(1-1), PBP2x118(1-1), PBP2x144(1-1) |
