# Supplementary figures and images for: Predicting β-lactam susceptibility from the genome of Streptococcus pneumoniae and other mitis group streptococci
Source: Front Microbiol. 2023 Mar 2;14:1120023. doi: 10.3389/fmicb.2023.1120023 (PMC10018206; doi:10.3389/fmicb.2023.1120023)

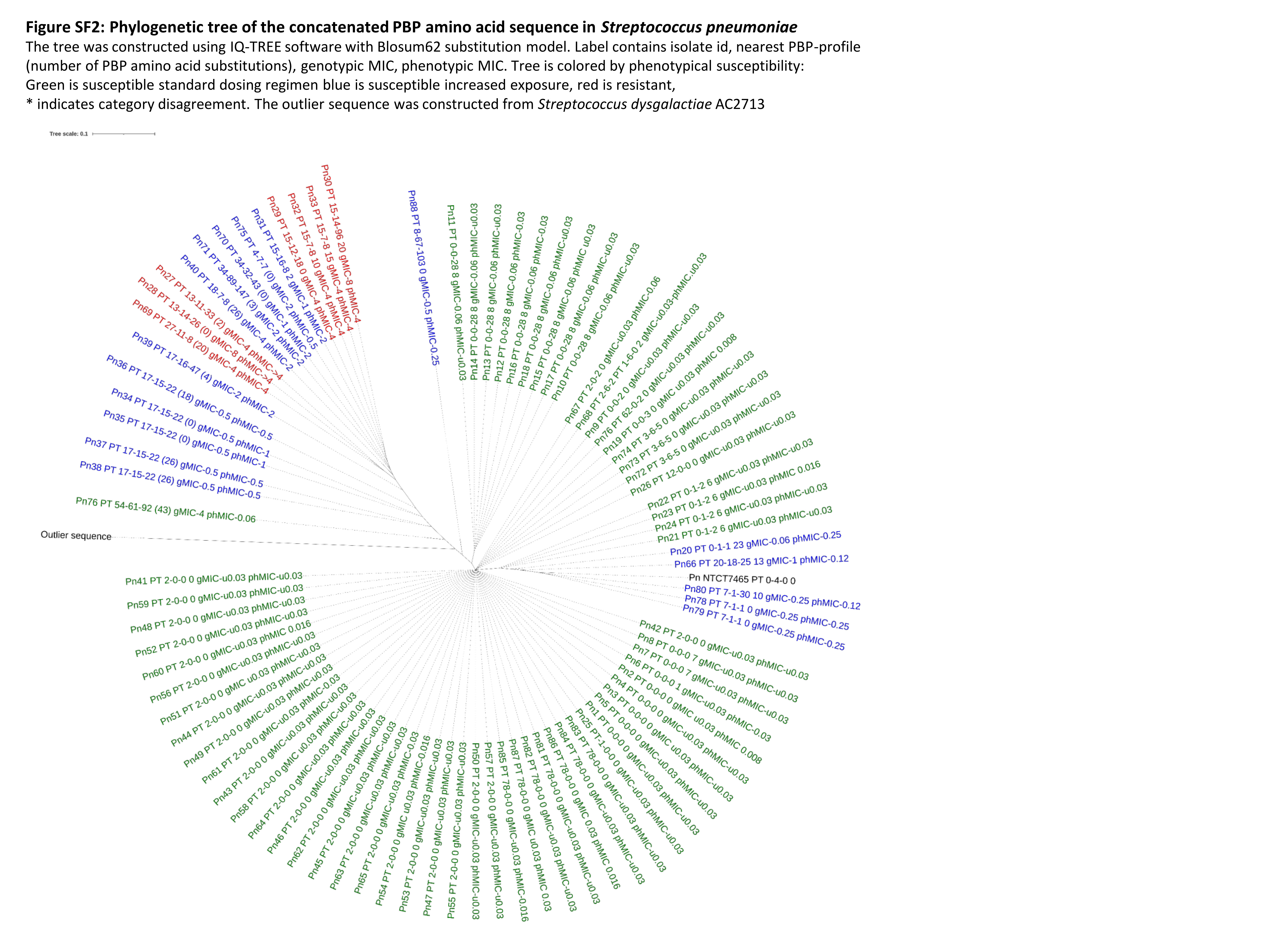

Supplement: Supplementary file 13 [file Image_2.TIF]

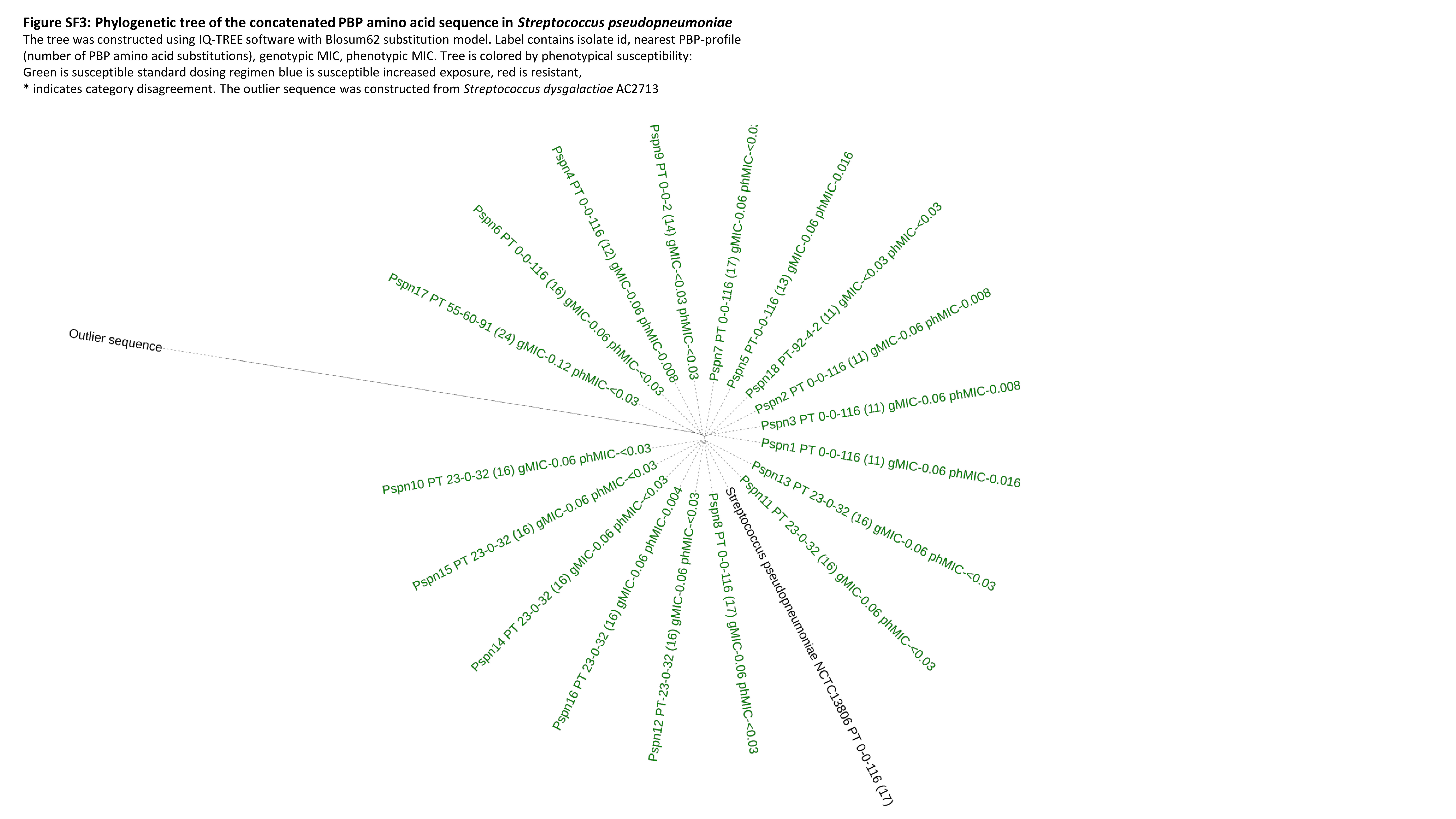

Supplement: Supplementary file 14 [file Image_3.TIF]

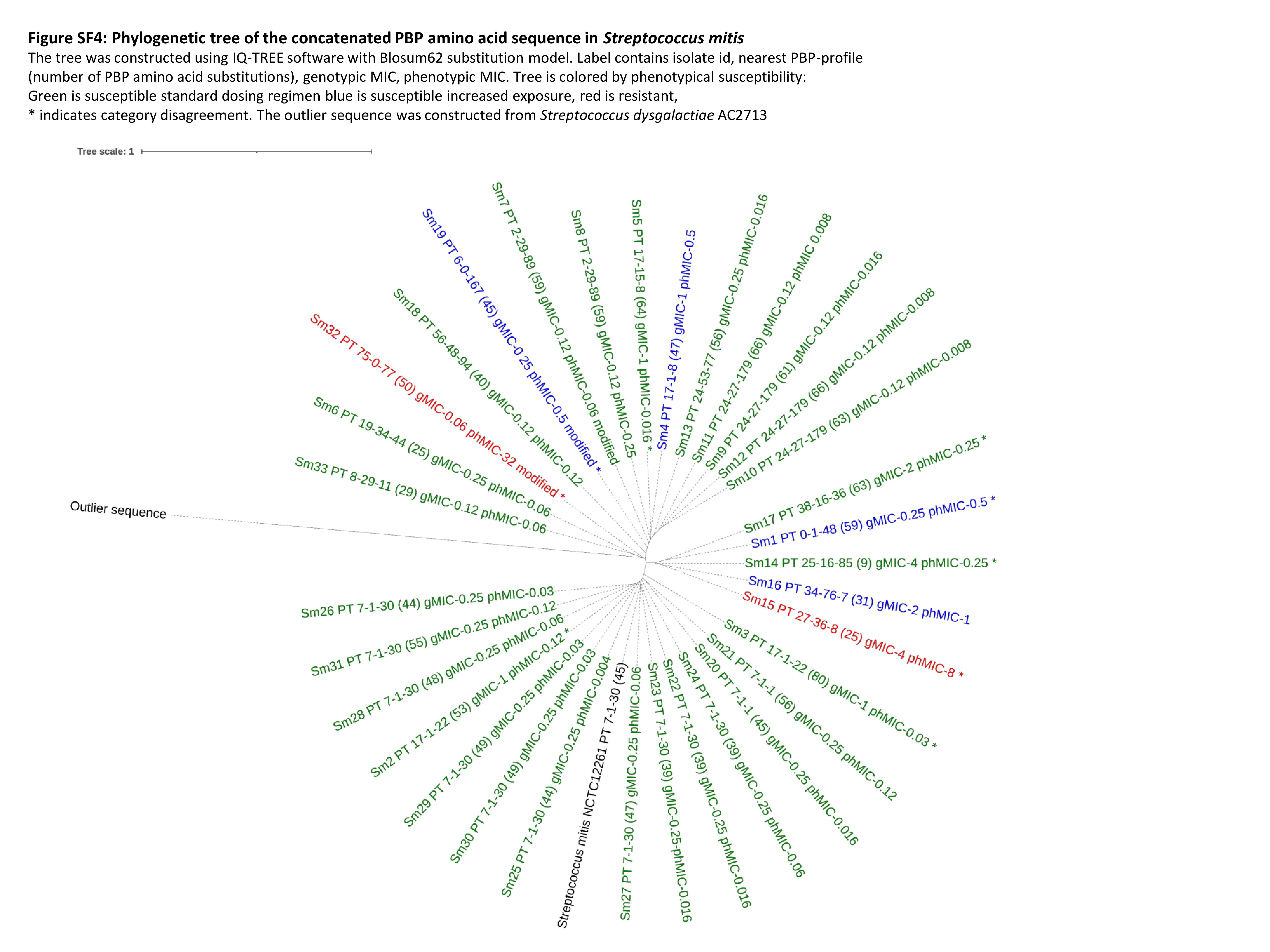

Supplement: Supplementary file 15 [file Image_4.TIF]

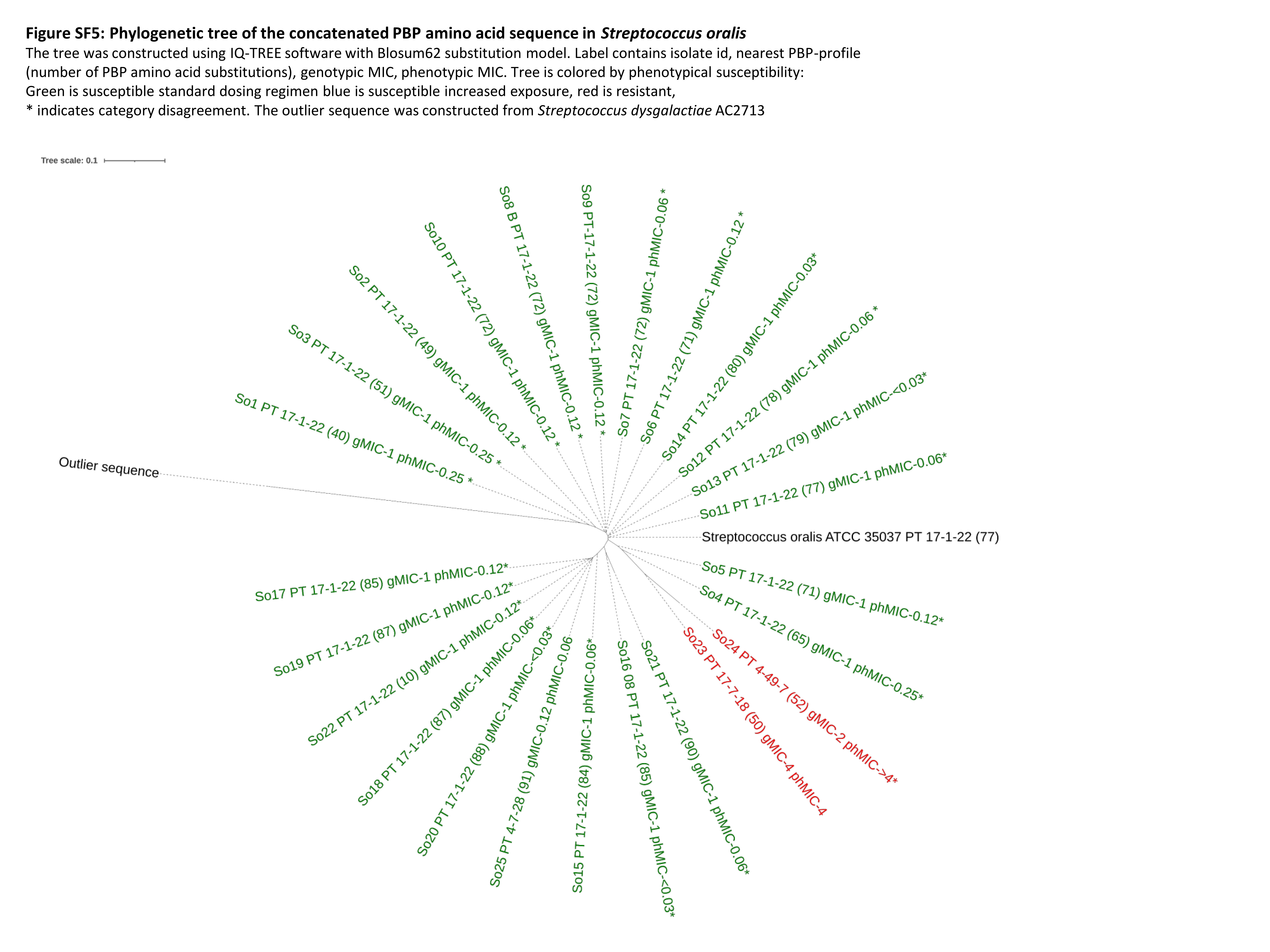

Supplement: Supplementary file 16 [file Image_5.TIF]
